# Ultrafast electron transfer at the In_2_O_3_/Nb_2_O_5_ S-scheme interface for CO_2_ photoreduction

**DOI:** 10.1038/s41467-024-49004-7

**Published:** 2024-06-05

**Authors:** Xianyu Deng, Jianjun Zhang, Kezhen Qi, Guijie Liang, Feiyan Xu, Jiaguo Yu

**Affiliations:** 1 Laboratory of Solar Fuel, Faculty of Materials Science and Chemistry, China University of Geosciences, Wuhan, 430078 PR China; 2 College of Pharmacy, Dali University, Dali, 671003 PR China; 3 Hubei Key Laboratory of Low Dimensional Optoelectronic Materials and Devices, Hubei University of Arts and Science, Xiangyang, 441053 PR China

**Keywords:** Photocatalysis, Photocatalysis, Catalytic mechanisms, Materials chemistry

## Abstract

Constructing S-scheme heterojunctions proves proficient in achieving the spatial separation of potent photogenerated charge carriers for their participation in photoreactions. Nonetheless, the restricted contact areas between two phases within S-scheme heterostructures lead to inefficient interfacial charge transport, resulting in low photocatalytic efficiency from a kinetic perspective. Here, In_2_O_3_/Nb_2_O_5_ S-scheme heterojunctions are fabricated through a straightforward one-step electrospinning technique, enabling intimate contact between the two phases and thereby fostering ultrafast interfacial electron transfer (<10 ps), as analyzed via femtosecond transient absorption spectroscopy. As a result, powerful photo-electrons and holes accumulate in the Nb_2_O_5_ conduction band and In_2_O_3_ valence band, respectively, exhibiting extended long lifetimes and facilitating their involvement in subsequent photoreactions. Combined with the efficient chemisorption and activation of stable CO_2_ on the Nb_2_O_5_, the resulting In_2_O_3_/Nb_2_O_5_ hybrid nanofibers demonstrate improved photocatalytic performance for CO_2_ conversion.

## Abstract (web summary)

In_2_O_3_/Nb_2_O_5_ S-scheme heterojunctions fabricated via a one-step electrospinning technique facilitate contact between two phases, fostering ultrafast interfacial electron transfer to prolong lifetimes and improve CO_2_ photoreduction performance.

## Introduction

Excessive emissions of carbon dioxide (CO_2_) into the atmosphere have disrupted the natural carbon cycle, leading to severe environmental consequences, particularly the exacerbation of the greenhouse effect^1–6^. In response to this urgent global issue, harnessing abundant, clean, and inexhaustible sunlight to convert CO_2_ into valuable solar fuels has emerged as a promising strategy^7–13^. However, the effectiveness of CO_2_ photoreduction is constrained by the challenging chemisorption and activation of CO_2_ molecules on catalysts, primarily due to the high dissociation energy of the C=O bond (~750 kJ mol^–1^)^14–19^. Therefore, the development of advanced photocatalysts proficient in activating CO_2_ has become a pivotal concern within the realm of photocatalytic CO_2_ reduction^20–26^. Niobium pentoxide (Nb_2_O_5_), a non-toxic solid oxide renowned for its high conduction band (CB) level and potent reduction capability, has recently gained significant attention in photocatalysis^27–33^. Preliminary density functional theory (DFT) calculations indicate that CO_2_ molecules adsorbed onto the Nb_2_O_5_ undergo changes in both bond lengths and angle compared to free ones. Additionally, the two oxygen atoms of CO_2_ can form chemical bonds with niobium atoms of Nb_2_O_5_, suggesting the potential of Nb_2_O_5_ for activating stable CO_2_ molecules during CO_2_ photoreduction. Nevertheless, unitary Nb_2_O_5_ exhibits poor photocatalytic performance resulting from sluggish electron/hole separation and charge transfer kinetics. Consequently, developing hybrid heterojunctions involving Nb_2_O_5_, capable of activating CO_2_, promoting charge carrier transfer kinetics, and separating them to improve reduction efficiency, remains a significant yet challenging endeavor.

**Fig. 1:**
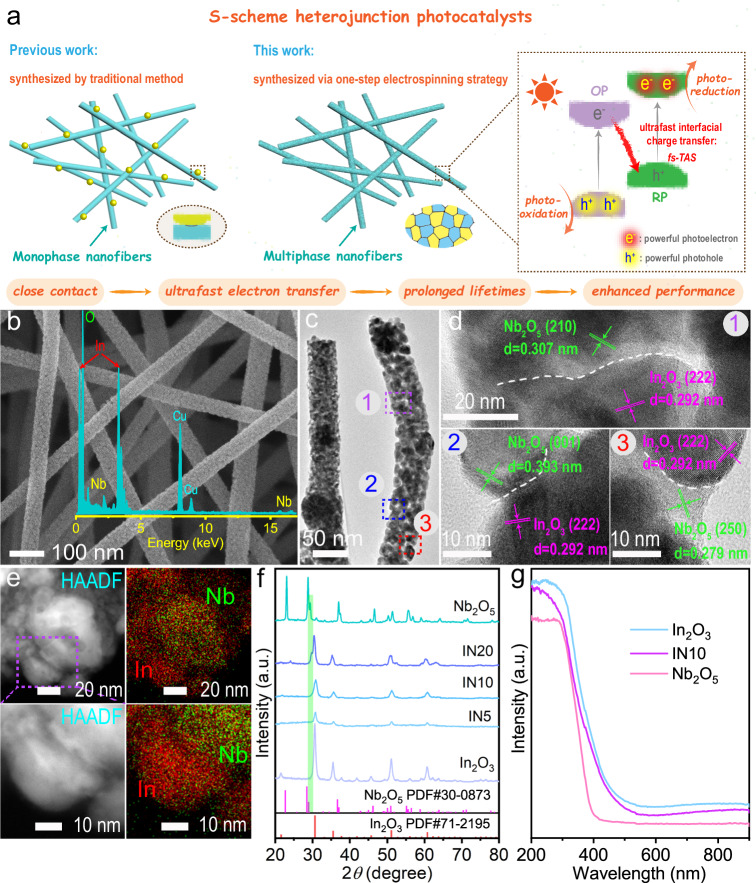
Morphology and structure of In_2_O_3_/Nb_2_O_5_ heterojunctions. **a** Schematic and the design concept of this study. OP and RP stand for oxidation photocatalyst and reduction photocatalyst, respectively. **b** FESEM image and EDX spectrum, (**c**) TEM image, and (**d**) HRTEM images of In_2_O_3_/Nb_2_O_5_ heterojunctions (IN10). **e** High-angle annular dark-field (HAADF) image and EDX elemental mappings of In and Nb elements in IN10 at different magnifications. **f** XRD patterns of In_2_O_3_, Nb_2_O_5_ and IN*x*. **g** UV-vis spectra of In_2_O_3_, Nb_2_O_5_, and IN10.

S-scheme heterojunctions, integrating both reduction and oxidation photocatalysts, have proven effective in spatially separating photogenerated charge carriers with robust redox capabilities^34–39^. Conventionally, constructing S-scheme heterojunctions involves initially acquiring photocatalyst I and subsequently applying photocatalyst II onto I through methods such as in situ growth or electrostatic self-assembly^40–42^. However, these post-hybridization methods cannot ensure the maximum contact area between the two phases at atomic levels, thus impeding the efficient interfacial transport of photogenerated carriers and compromising photocatalytic efficiency (Fig. 1a)^43^. In this study, we designed an S-scheme heterojunction by coupling Nb_2_O_5_ with indium oxide (In_2_O_3_), an oxidation photocatalyst with a narrow bandgap (~2.9 eV) and visible light absorption^44–51^. By mixing the precursors of both phases in the same electrospinning solution, In_2_O_3_ and Nb_2_O_5_ are simultaneously formed during the high-temperature calcination of the electrospun nanofibers. This “one-pot” preparation method ensures maximum phase contact without any hindrance, providing an unimpeded transport route and promoting interfacial charge transfer between In_2_O_3_ and Nb_2_O_5_ (Fig. 1a). Analysis using femtosecond transient absorption spectroscopy (fs-TAS) revealed ultrafast photoelectron transfer from the In_2_O_3_ CB to the Nb_2_O_5_ valence band (VB), inhibiting self-carrier recombination, effectively segregating powerful photoelectrons in the Nb_2_O_5_ CB and the holes in the In_2_O_3_ VB, as well as prolonging the long-lifetimes of the nanohybrids. Also benefiting from the chemisorption and activation of CO_2_ molecules on the catalyst, the resulting In_2_O_3_/Nb_2_O_5_ heterojunctions demonstrated enhanced performance in CO_2_ photoreduction. This work provides insights into ultrafast charge transfer at the S-scheme heterojunction interface through fs-TAS investigations, offering an essential understanding for the development of heterojunctions and broadening their potential applications in artificial photosynthesis.

## Results and discussion

### Characterizations and charge separation mechanism of In_2_O_3_/Nb_2_O_5_ heterojunctions

The In_2_O_3_/Nb_2_O_5_ heterojunctions, synthesized through a one-step electrospinning procedure, are designated as IN*x*, where I and N represent In_2_O_3_ and Nb_2_O_5_, respectively, and *x* signifies the weight percentage of Nb_2_O_5_ relative to In_2_O_3_. The precise Nb_2_O_5_ content of all composites was determined using inductively coupled plasma-atomic emission spectrometry (ICP-AES), and the results are presented in Supplementary Table 1. Field emission scanning electron microscopy (FESEM) images reveal distinctive morphologies: pure In_2_O_3_ exhibits a fibrous structure, while pristine Nb_2_O_5_ displays a clubbed pattern (Supplementary Fig. 1). The In_2_O_3_/Nb_2_O_5_ heterojunction (IN10) shows a uniform fibrous morphology with a rough surface and diameters below 100 nm, as observed via both FESEM and transmission electron microscopy (TEM) (Fig. 1b, c). High-resolution TEM (HRTEM) images of IN10 reveal discernible two-phase grain boundaries with lattice fringes corresponding to In_2_O_3_ and Nb_2_O_5_, respectively (Fig. 1d). The random distribution of In_2_O_3_ and Nb_2_O_5_ nanoparticles within the nanofibers ensures close contact, facilitating unimpeded interfacial transport and efficient separation of photoexcited charge carriers (as discussed below). Energy-dispersive X-ray (EDX) analysis (inset in Fig. 1b) and elemental mappings of IN10 (Supplementary Fig. 2) unambiguously confirm the presence of In, Nb, and O elements within the nanohybrid, offering compelling evidence for the formation of In_2_O_3_/Nb_2_O_5_ heterojunctions. Figure 1e displays enlarged elemental mappings targeting a grain-to-grain area within IN10. It is apparent that the distribution of Nb and In elements exhibits a non-overlapping pattern along the interface of the two phases. X-ray diffraction (XRD) patterns of pure In_2_O_3_ nanofibers (Fig. 1f) indicate the monoclinic phase (PDF#71-2195), while characteristic peaks corresponding to Nb_2_O_5_ (PDF#30-0873) emerge in the IN*x* composites at 20 wt.% Nb_2_O_5_ content, signifying the successful synthesis of In_2_O_3_/Nb_2_O_5_ heterojunctions. UV-vis diffuse reflectance spectroscopy (DRS) delineates the optical properties of In_2_O_3_, Nb_2_O_5_, and In_2_O_3_/Nb_2_O_5_ composites (Fig. 1g). The absorption edges of pristine In_2_O_3_ and Nb_2_O_5_ are positioned at 425 and 390 nm, corresponding to bandgaps of 2.9 and 3.2 eV, respectively. Compared to pure Nb_2_O_5_, the slightly improved UV and visible light absorption characteristics of IN10 suggest successful hybridization due to the strong light absorption capacity of In_2_O_3_.

**Fig. 2:**
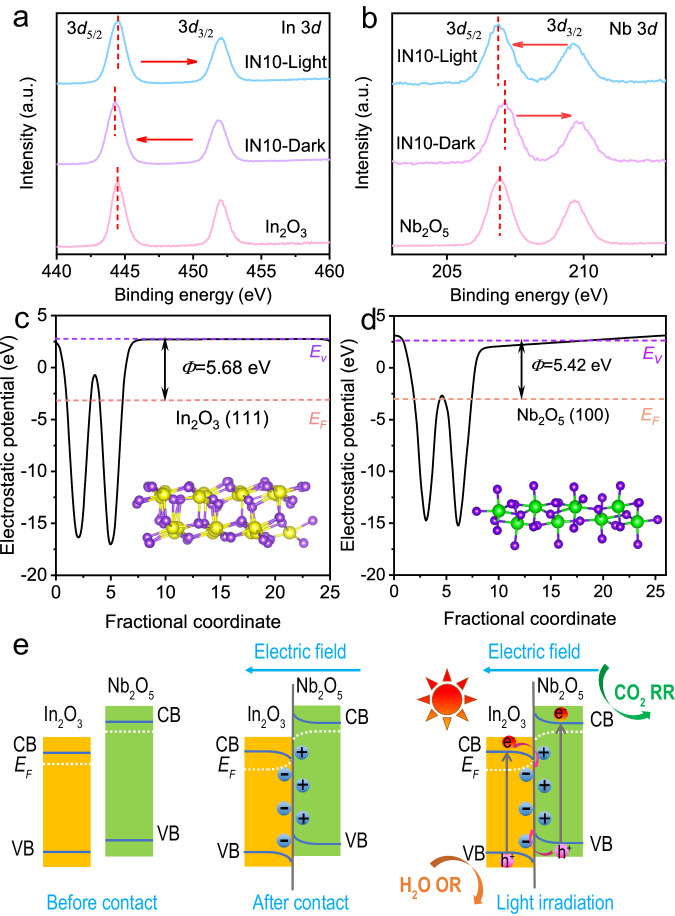
Electron transfer between In_2_O_3_ and Nb_2_O_5_ within the heterojunctions. The high-resolution XPS spectra of (**a**) In 3*d*, and (**b**) Nb 3*d* of In_2_O_3_, Nb_2_O_5_, and IN10. Calculated electrostatic potentials of (**c**) In_2_O_3_ (111) and (**d**) Nb_2_O_5_ (100) slabs. The yellow, green, and purple spheres represent In, Nb, and O atoms, respectively. **e** The formation of In_2_O_3_/Nb_2_O_5_ S-scheme heterojunction, and the proposed charge transfer and separation mechanism. RR and OR stand for reduction reaction and oxidation reaction, respectively.

X-ray photoelectron spectroscopy (XPS) was utilized to analyze the surface chemical states and compositions of the resulting samples. The survey spectrum of IN10 reveals the presence of In, Nb, and O elements in the hybrid nanofibers (Supplementary Fig. 3). In the high-resolution In 3*d* XPS spectra (Fig. 2a), two distinct peaks are observed at 444.5 and 452.0 eV, corresponding to the 3*d*_5/2_ and 3*d*_3/2_ states of trivalent In^3+^ within In_2_O_3_, respectively. The signals associated with Nb 3*d*_5/2_ and Nb 3*d*_3/2_ appear at 206.9 and 209.7 eV, respectively, confirming the existence of pentavalent Nb^5+^ in the samples (Fig. 2b)^30^. The O 1 *s* XPS spectra of In_2_O_3_, IN10, and Nb_2_O_5_ (Supplementary Fig. 4a–c) consistently exhibit peaks attributed to lattice oxygen and surface hydroxyls (-OH). Notably, in the IN10 composite, the binding energies (BEs) of In 3*d* show negative shifts compared to pure In_2_O_3_, while the peaks of Nb 3*d* shift towards higher BEs in comparison with bare Nb_2_O_5_. These shifts suggest that electrons are transferred from Nb_2_O_5_ to In_2_O_3_ upon contact, indicating the creation of a directional interfacial electric field (IEF) from Nb_2_O_5_ to In_2_O_3_, and simultaneously leading to the bending of the energy bands at the interfaces. To further substantiate the electron transfer process between In_2_O_3_ and Nb_2_O_5_, the work function (*Φ*) was determined through DFT simulations by calculating the energy difference between the vacuum and Fermi levels, based on the electrostatic potential of the materials. As illustrated in Fig. 2c, d and Supplementary Figs. 5, 6, the estimated *Φ* value of In_2_O_3_ (111) is larger than that of Nb_2_O_5_ (100), with both facets exhibiting the lowest surface energy (Supplementary Tables 2 and 3). Consequently, Nb_2_O_5_ possesses a higher Fermi level (*E*_F_) than In_2_O_3_, promoting the transfer of electrons from Nb_2_O_5_ to In_2_O_3_ until reaching the same *E*_F_ at the interface (Fig. 2e). These analyses align with the aforementioned XPS results and contribute to the efficient separation of photogenerated charge carriers (as discussed below).

To investigate the photoinduced charge transfer mechanism of the In_2_O_3_/Nb_2_O_5_ heterojunctions, the band structure of In_2_O_3_ and Nb_2_O_5_ was first studied. According to the ultraviolet photoelectron spectroscopy (UPS) spectra (Supplementary Fig. 7a, b), the VB maximum of In_2_O_3_ and Nb_2_O_5_ is estimated at 2.41 and 2.28 V (vs. standard hydrogen electrode, SHE), respectively. Combined with the bandgap values disclosed in Supplementary Fig. 7c, the CB minimum is established as –0.49 V for In_2_O_3_ and –0.96 V for Nb_2_O_5_ (Supplementary Fig. 7d)^33^. Based on the previous discussion involving XPS and DFT results, it is evident that the *E*_F_ of Nb_2_O_5_ is higher than that of In_2_O_3_, which induces the migration of electrons from Nb_2_O_5_ to In_2_O_3_, leading to the creation of an IEF at the interface, as well as band alignment upon contact. Under light irradiation, electrons in the In_2_O_3_ and Nb_2_O_5_ VBs are initially excited to their respective CBs. Due to the bent energy bands, the IEF with the direction from Nb_2_O_5_ to In_2_O_3_, and the Coulomb attraction between electrons and holes, the photogenerated electrons in the In_2_O_3_ CB tend to transfer to the Nb_2_O_5_ VB and recombine with its holes. These consumed photoelectrons and photoholes are characterized by their weak reduction and oxidation capacities. As a result, photogenerated charge carriers with strong redox capabilities within the Nb_2_O_5_ CB and the In_2_O_3_ VB undergo separation and preservation, actively participating in subsequent photoreactions. This charge transfer pathway implies the formation of an S-scheme heterojunction between In_2_O_3_ and Nb_2_O_5_, visually depicted in Fig. 2e.

In situ irradiated XPS was conducted to verify the S-scheme charge transfer route within the In_2_O_3_/Nb_2_O_5_ heterojunctions. As shown in Fig. 2a, b, upon exposure to light, the BEs of In 3*d* in IN10 display notable positive shifts, while the Nb 3*d* peaks shift towards lower BEs, with respect to those in the dark. These observed BE shifts provide strong evidence for the transfer of photogenerated electrons from In_2_O_3_ to Nb_2_O_5_, thus corroborating the proposed S-scheme photocatalytic mechanism. The efficiency of charge separation in the In_2_O_3_/Nb_2_O_5_ S-scheme heterojunctions was assessed through steady-state photoluminescence (PL) and photoelectrochemical measurements. The PL emission intensity of the IN10 composite is weaker than both pure In_2_O_3_ and Nb_2_O_5_ (Supplementary Fig. 8), indicating a significant inhibition of the electron/hole recombination within the In_2_O_3_/Nb_2_O_5_ S-scheme hybrid nanofibers. Moreover, during the long-term photoelectrochemical test, IN10 consistently exhibits the highest and most stable photocurrent density in contrast to pristine In_2_O_3_ and Nb_2_O_5_ (Supplementary Fig. 9), underscoring the efficient charge separation within the In_2_O_3_/Nb_2_O_5_ nanohybrids. Electrochemical impedance spectroscopy (EIS) results demonstrate that IN10 displays a smaller arc radius in the Nyquist plot compared to bare In_2_O_3_ and Nb_2_O_5_ (Supplementary Fig. 10), signifying a lower charge transfer resistance in the In_2_O_3_/Nb_2_O_5_ composite. These analyses collectively confirm that the hybridization of In_2_O_3_ and Nb_2_O_5_ to form S-scheme heterojunctions can boost charge transfer and effectively reduce the electron/hole recombination, thus facilitating high-efficiency photocatalytic CO_2_ reduction^52–56^.

The accumulation of photogenerated electrons and holes after S-scheme charge separation was explored through electron paramagnetic resonance (EPR) spectroscopy. The reduction potential of 5,5-dimethyl-1-pyrroline N-oxide (DMPO)-•O_2_^−^ and the oxidation potential of DMPO-•OH are −0.74 and 2.28 V (vs. SHE), respectively. Compared to pristine In_2_O_3_ or Nb_2_O_5_, the In_2_O_3_/Nb_2_O_5_ composite shows intensive EPR signals for both •O_2_^−^ and •OH radicals (Supplementary Fig. 11). This observation signifies the efficient separation and accumulation of energetic photoelectrons in the Nb_2_O_5_ CB and photoholes in the In_2_O_3_ VB, providing compelling evidence for the S-scheme charge separation mechanism.

### Ultrafast electron transfer at the In_2_O_3_/Nb_2_O_5_ S-scheme heterojunction interface

**Fig. 3:**
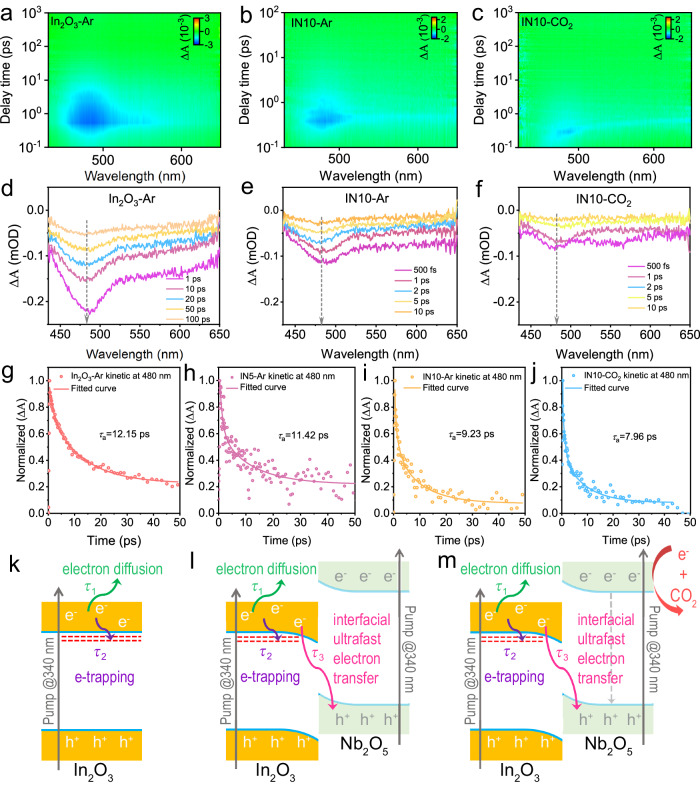
Insights into charge transfer dynamics in pure In_2_O_3_ and In_2_O_3_/Nb_2_O_5_ S-scheme heterojunctions. The pseudocolor plots and transient absorption spectra recorded at indicated delay times measured with 340 nm excitation: (**a**, **d**) pure In_2_O_3_ in Ar, (**b**, **e**) IN10 in Ar, and (**c**, **f**) IN10 in CO_2_. Corresponding kinetic decay curves at 480 nm within 50 ps: **g** pure In_2_O_3_ in Ar, (**h**) IN5 in Ar, (**i**) IN10 in Ar, and (**j**) IN10 in CO_2_. The decay pathways of photogenerated electrons in (**k**) pure In_2_O_3_, **l** In_2_O_3_/Nb_2_O_5_ heterojunctions in Ar, and (**m**) In_2_O_3_/Nb_2_O_5_ heterojunctions in CO_2_.

Fs-TAS was employed to delve deeper into the dynamics of photoelectron transfer at the In_2_O_3_/Nb_2_O_5_ S-scheme interface. As depicted in Fig. 3a–f, both pristine In_2_O_3_ and the In_2_O_3_/Nb_2_O_5_ heterojunctions (IN5 and IN10) exhibit noticeable negative peaks at ~480 nm when excited at 340 nm, which correspond to the ground state bleaching (GSB) signals of In_2_O_3_ and provide insights into the population of photoelectrons in its CB^57^. This assignment was further supported by an experiment using AgNO_3_ as an electron scavenger, wherein the signal virtually disappears (Supplementary Fig. 12), indicating that the photogenerated electrons are trapped by the scavenger, leaving no electrons to recombine with the holes. The normalized recovery kinetics of pristine In_2_O_3_ at 480 nm, monitored within 50 ps, were fitted with a two-exponential function (Fig. 3g and Supplementary Table 4), assigning to the interband diffusion (Process I) and the trapping by shallow trap states (Process II) of the photogenerated electrons in the In_2_O_3_ CB. Upon integrating In_2_O_3_ with Nb_2_O_5_, an additional ultrafast pathway (<10 ps) emerges for the photoelectrons in the In_2_O_3_ CB, namely, their transfer to the Nb_2_O_5_ VB (Process III)^58^. Notably, in an Ar atmosphere, both *τ*_1_ and *τ*_2_ lifetimes demonstrate a gradual decrease with increasing Nb_2_O_5_ content (IN5 and IN10, Fig. 3h, i and Supplementary Table 4). This implies the rapid migration of more photoelectrons from the In_2_O_3_ CB to Nb_2_O_5_ upon hybridization, resulting in fewer electrons available for diffusion and trapping processes (Fig. 3k, l). Under a CO_2_ atmosphere (Fig. 3j, m), photoelectrons in the Nb_2_O_5_ CB react with CO_2_ molecules, accelerating the transfer of more photoelectrons from the In_2_O_3_ CB to the Nb_2_O_5_ VB to recombine with its photoholes, thereby shortening the lifetimes. The fs-TAS analysis of a physically-mixed composite of In_2_O_3_ and Nb_2_O_5_ reveals longer lifetimes (*τ*_1_ and *τ*_2_) compared to In_2_O_3_/Nb_2_O_5_ heterostructures (Supplementary Fig. 13), indicating inefficient electron transfer from In_2_O_3_ to Nb_2_O_5_ and underscoring the advantages of interfacial phase contact within the S-scheme heterojunction for efficient charge transfer and separation.

**Fig. 4:**
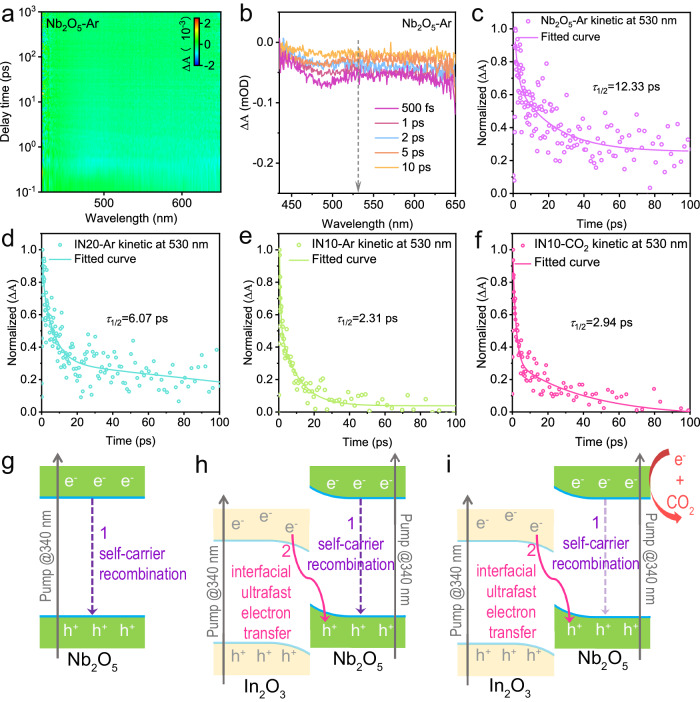
Insights into charge transfer dynamics in pure Nb_2_O_5_ and In_2_O_3_/Nb_2_O_5_ S-scheme heterojunctions. **a** The pseudocolor plot, and (**b**) transient absorption spectra of pure Nb_2_O_5_ recorded at indicated delay times measured with 340 nm excitation. Corresponding kinetic decay curves at 530 nm within 100 ps of (**c**) pure Nb_2_O_5_ in Ar, (**d**) IN20 in Ar, (**e**) IN10 in Ar, and (**f**) IN10 in CO_2_. The decay pathways of photogenerated holes in (**g**) pure Nb_2_O_5_, (**h**) In_2_O_3_/Nb_2_O_5_ heterojunctions in Ar, and (**i**) In_2_O_3_/Nb_2_O_5_ heterojunctions in CO_2_.

On the other hand, the broad GSB signal of Nb_2_O_5_ appears around 500 nm (Fig. 4a, b), corresponding to the population of photoholes in its VB as affirmed by the disappearance of the signal after introducing a hole-trapping agent (lactic acid) (Supplementary Fig. 14). To minimize the interference of In_2_O_3_, kinetic decay curves of bare Nb_2_O_5_ and the In_2_O_3_/Nb_2_O_5_ nanohybrids (IN20 and IN10) are derived at 530 nm (Fig. 4c–e), which involve two processes related to photoexcited holes in the Nb_2_O_5_ VB, i.e., recombination with self-generated photoelectrons (process 1) and recombination with photoelectrons transferred from In_2_O_3_ (process 2) (Fig. 4g). Under an Ar atmosphere, the half-life (*τ*_1/2_) of both IN20 and IN10 is shorter than that of pristine Nb_2_O_5_, signifying the migration of photoelectrons from the In_2_O_3_ CB to Nb_2_O_5_ and thereby reducing the number of VB photoholes (Fig. 4h). In addition, the In_2_O_3_/Nb_2_O_5_ heterojunctions exhibit a composition-dependent half-life, with a shorter value as Nb_2_O_5_ content decreases (IN10 < IN20). According to the S-scheme charge separation mechanism, photoelectrons in the In_2_O_3_ CB transfer to the Nb_2_O_5_ VB and recombine with its photoholes in equal proportions. At an ideal In_2_O_3_/Nb_2_O_5_ ratio (i.e., IN10), the population of photogenerated charge carriers is approximatively identical in both materials, leaving few excess photoholes in the Nb_2_O_5_ VB and consequently shortening the lifetime after S-scheme charge separation. When the Nb_2_O_5_ content deviates from its optimal value (i.e., IN20), some excessive photoholes remain in the Nb_2_O_5_ VB, leading to a longer lifetime than IN10. Under a CO_2_ atmosphere, photoelectrons in the Nb_2_O_5_ CB actively react with CO_2_, diminishing their recombination with holes while facilitating the photoelectron transfer from the In_2_O_3_ CB to the Nb_2_O_5_ VB, thus resulting in no notable alteration in the lifetime (Fig. 4i). The analyses emphasize the ultrafast electron transfer at the In_2_O_3_/Nb_2_O_5_ S-scheme heterojunction interface for suppressing self-carrier recombination and spatially separating photoelectrons in the Nb_2_O_5_ CB and photoholes in the In_2_O_3_ VB.

Time-resolved fluorescence spectroscopy (TRPL) was conducted to investigate the long lifetime of the photocatalysts. As presented in Supplementary Fig. 15, under an Ar atmosphere, the IN10 hybrid exhibits a longer average lifetime (*τ*_a_) with respect to pristine In_2_O_3_ and Nb_2_O_5_ at an emission wavelength of 470 nm, where the fluorescence signals originate from both In_2_O_3_ and Nb_2_O_5_. Following the proposed S-scheme mechanism for IN10, photoelectrons in the In_2_O_3_ CB migrate to the Nb_2_O_5_ VB and recombine with its photoholes, resulting in the accumulation of powerful electrons in the Nb_2_O_5_ CB and holes in the In_2_O_3_ VB, thereby prolonging the charge carrier lifetimes. The physically-mixed composite of In_2_O_3_ and Nb_2_O_5_ displays a shorter *τ*_a_ than the In_2_O_3_/Nb_2_O_5_ heterojunction, highlighting the significance of ultrafast interfacial electron transfer in extending carrier lifetimes. Furthermore, in situ TRPL was employed to explore the relationship between the ultrafast charge transfer-induced carrier lifetimes and the photocatalytic performance. The *τ*_a_ of IN10 recorded under a CO_2_ atmosphere is shorter than that under an Ar atmosphere (Supplementary Fig. 16a), suggesting that a substantial portion of photogenerated electrons in the Nb_2_O_5_ CB is involved in CO_2_ photoreduction, thereby leaving fewer charge carriers available for recombination. Bare In_2_O_3_, Nb_2_O_5_, and their physically-mixed composite (Supplementary Fig. 16b–d) reveal almost identical decay curves under both CO_2_ and Ar atmospheres, indicating their poor photoreaction performance. Overall, the ultrafast interfacial charge transfer within the In_2_O_3_/Nb_2_O_5_ heterojunctions plays triple roles: preventing the recombination of self-carriers, separating powerful photoelectrons and photoholes, and extending their long lifetimes.

### Chemisorption, activation and photoreduction of CO_2_ over In_2_O_3_/Nb_2_O_5_ hybrid nanofibers

**Fig. 5:**
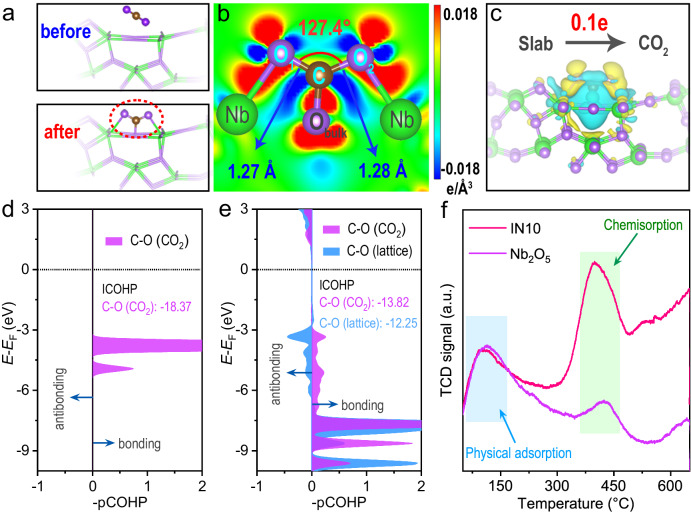
Chemisorption and activation of CO_2_ over In_2_O_3_/Nb_2_O_5_ hybrid nanofibers. **a**, **b** Optimized structures, and (**c**) the corresponding charge density difference image of CO_2_ adsorbed on the Nb_2_O_5_; cyan and yellow regions represent electron depletion and accumulation, respectively. The isosurface level is set to 0.002 e Å^–3^. Projected COHP profiles of (**d**) free CO_2_ and (**e**) adsorbed CO_2_ molecules. **f** CO_2_-TPD spectra of pure Nb_2_O_5_ and IN10 composite.

The adsorption and activation of CO_2_ molecules on the catalyst, pivotal steps for CO_2_ photoreaction, were investigated using DFT simulations and CO_2_-temperature programmed desorption (TPD) analysis. Upon CO_2_ adsorption on Nb_2_O_5_, distinct chemisorption processes occur, as evident from several observations (Fig. 5a–c and Supplementary Fig. 17): (i) a pronounced bending of the O=C=O bond at an angle of 127.4°; (ii) elongation of the bond length compared to free CO_2_ molecule (1.16 Å); (iii) formation of new bonds between CO_2_ and Nb_2_O_5_; and (iv) transfer of electrons from Nb_2_O_5_ to CO_2_ (Supplementary Table 5). The integrated crystal orbital Hamiltonian population (ICOHP) of the C-O pairs is –18.37 and –13.82 eV in free and adsorbed CO_2_, respectively, signifying CO_2_ activation over Nb_2_O_5_. Moreover, the formation of new C-O (lattice) bonds with an ICOHP of –12.25 eV provides additional compelling evidence of the robust CO_2_ chemisorption on Nb_2_O_5_ (Fig. 5d, e). The CO_2_-TPD profiles for Nb_2_O_5_ and IN10 (Fig. 5f) demonstrate the desorption of physiosorbed CO_2_ at 70–150 °C. At a high temperature ranging from 370 to 450 °C, both samples exhibit prominent desorption signals indicative of CO_2_ chemisorption on the catalysts. The CO_2_ adsorption energy (*E*_ads_) on the Nb atom is more negative than that on the O atom (Supplementary Fig. 18 and Table 6), suggesting that Nb atoms serve as active sites for the chemisorption and activation of CO_2_ during photoreduction.

**Fig. 6:**
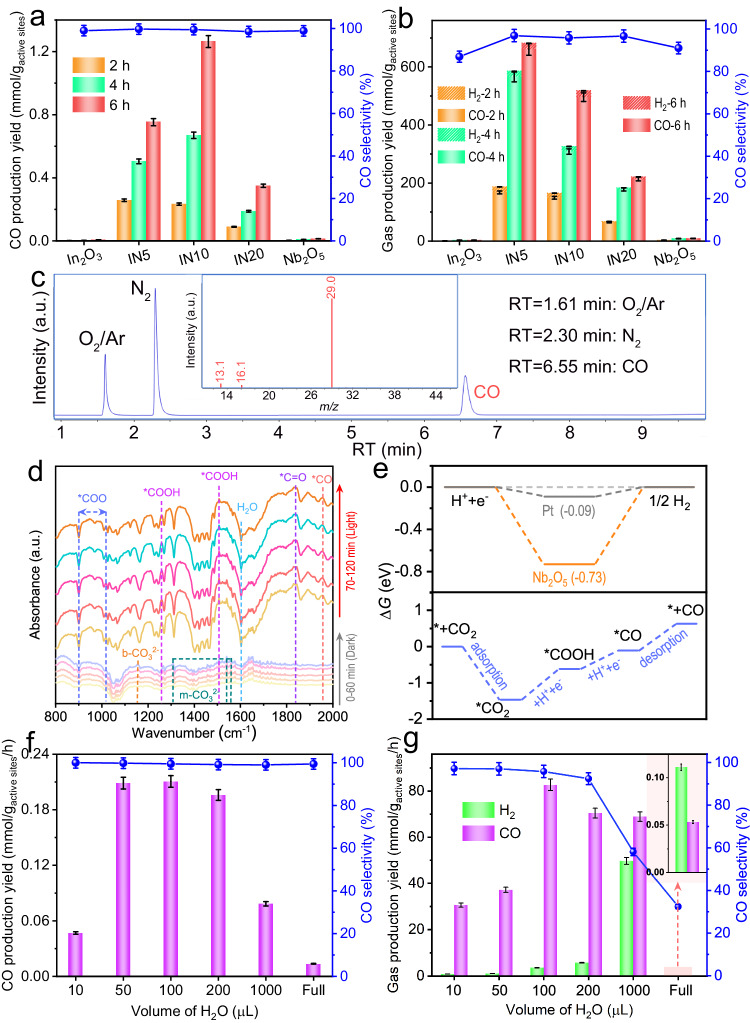
Performance and mechanism insights into photocatalytic CO_2_ reduction. The production yields and CO selectivity over In_2_O_3_, IN*x,* and Nb_2_O_5_ during six-hour experiments conducted under UV-visible light irradiation: **a** without any molecule cocatalyst or scavenger, (**b**) with [Ru^II^(bpy)_3_]Cl_2_·6H_2_O and BIH. **c** The total ion chromatography and the corresponding mass spectra of the products in the photocatalytic reduction of ^13^CO_2_ over IN10. **d** In situ DRIFT spectra for the photocatalytic CO_2_ reduction over IN10. **e** Gibbs free energy diagrams of CO_2_ photoreduction and H_2_ production over Nb_2_O_5_ (100) slab. The influence of H_2_O volumes on product selectivity over IN10: **f** without any molecule cocatalyst or scavenger, (**g**) with [Ru^II^(bpy)_3_]Cl_2_·6H_2_O and BIH. The error bars (mean ± standard deviation) were obtained based on three independent photocatalytic experiments.

The photocatalytic activities for CO_2_ reduction of In_2_O_3_, IN*x,* and Nb_2_O_5_ were assessed in an online closed gas-circulation system (OLPCRS-2, Shanghai Boyi Scientific Instrument Co., Ltd., Supplementary Fig. 19) equipped with a glass reaction cell. Blank control experiments affirm that the concurrent presence of photocatalysts, CO_2_, H_2_O, and light irradiation is essential for initiating the photoreaction (Supplementary Fig. 20). In the absence of any molecule cocatalyst or scavenger, all the samples yielded CO as the reduction product with nearly 100% selectivity (Fig. 6a). Pure In_2_O_3_ and Nb_2_O_5_ exhibit poor photocatalytic performance due to the rapid recombination of photogenerated carriers inherent in single photocatalysts. However, the integration of In_2_O_3_ with Nb_2_O_5_ enhances CO_2_ photoreduction activities, leading to a maximum CO production yield of 0.21 mmol g_active sites_^–1^ h^–1^ over the IN10 composite. A comparison of CO_2_ photoreduction performance was conducted among the In_2_O_3_/Nb_2_O_5_ hybrid nanofibers, the In_2_O_3_/Nb_2_O_5_ nanohybrid synthesized via the traditional dip-calcination method, and a physically-mixed composite of In_2_O_3_ and Nb_2_O_5_. This comparison emphasizes the critical importance of intimate interface contact between the two phases for facilitating ultrafast interfacial electron transfer within the S-scheme heterojunction (Supplementary Fig. 21). Upon introducing tris(2,2′-bipyridyl)ruthenium(II) chloride hexahydrate ([Ru^II^(bpy)_3_]Cl_2_·6H_2_O) and 1,3-dimethyl-2-phenyl-2,3-dihydro-1H-benzo[d]imidazole (BIH) as the molecular catalyst and hole scavenger, respectively, a substantial amount of CO and a minor quantity of H_2_ were detected, with the highest production yields (109.6 mmol g_active sites_^–1^ h^–1^ for CO and 3.5 mmol g_active sites_^–1^ h^–1^ for H_2_) observed over the In_2_O_3_/Nb_2_O_5_ nanohybrid (Fig. 6b). To elucidate the origin of the photoreduction product, isotope-labeled carbon dioxide (^13^CO_2_) was employed as the substitute source gas for photocatalytic CO_2_ reduction over IN10. Distinct peaks observed at 1.61 and 2.30 min in the total ion chromatography are assigned to O_2_/Ar and N_2_, respectively (Supplementary Fig. 22). Another prominent peak emerges at ~6.55 min, corresponding to CO, which generates the predominant mass spectrometry signal at *m/z* = 29 (^13^CO), accompanied by two additional fragments at *m/z* = 13 and 16 (^13^C and O) (Fig. 6c). Additionally, thermogravimetric (TGA) analyses of pure In_2_O_3_, pristine Nb_2_O_5_, and the In_2_O_3_/Nb_2_O_5_ nanohybrid (IN10) reveal no perceptible weight changes up to 800 °C (Supplementary Fig. 23), suggesting that no carbon residual remains in the samples after a 2-h calcination at 600 °C. These findings confirm that the reduction product originates solely from the input CO_2_, ruling out other potential carbon sources^59^. The recyclability and stability of IN10 for CO_2_ photoreduction are confirmed, demonstrating a negligible decline in production yields over four cycles (Supplementary Fig. S24). The XRD pattern (Supplementary Fig. 25) and the In 3*d* and Nb 3*d* XPS spectra (Supplementary Fig. 26) of IN10 after the photoreaction exhibit inconspicuous changes compared to the fresh one, suggesting the photostability of the In_2_O_3_/Nb_2_O_5_ heterojunctions.

The reaction mechanism of CO_2_ photoreduction was explored using both in situ diffuse reflectance infrared Fourier transform spectroscopy (DRIFTS, Supplementary Fig. 27) and DFT calculations. As depicted in Fig. 6d, the presence of bidentate carbonate (b-CO_3_^2–^) and monodentate carbonate (m-CO_3_^2–^) after the introduction of CO_2_ into the system in the dark manifests the chemisorption of CO_2_ on IN10. Under light irradiation, new adsorption bands of *COOH (1258 and 1507 cm^–1^), *COO (carboxyl, 900 and 1017 cm^–1^), *C=O (carbonyl, 1839 cm^–1^), and *CO (absorbed CO, 1956 cm^–1^) are detected, which are key intermediates in the conversion of CO_2_ to CO. In light of these observations, the pathway for CO_2_ photoreduction over In_2_O_3_/Nb_2_O_5_ hybrid nanofibers is proposed as follows, where * denotes the reaction active site^23,60,61^:

1 $$ \ast+{{{{{{\rm{CO}}}}}}}_{2}\to*{{{{{{\rm{CO}}}}}}}_{2}$$

2 $$*{{{{{{\rm{CO}}}}}}}_{2}+{{{{{{\rm{H}}}}}}}^{+}+{{{{{{\rm{e}}}}}}}^{{{{{{-}}}}}}\to*{{{{{\rm{COOH}}}}}}$$

3 $$*{{{{{\rm{COOH}}}}}}+{{{{{{\rm{H}}}}}}}^{+}+{{{{{{\rm{e}}}}}}}^{{{{{{-}}}}}}\to*{{{{{\rm{CO}}}}}}+{{{\rm{H}}}_{2}{O}}$$

4 $$*{{{{{\rm{CO}}}}}}\to \ast+{{{{{\rm{CO}}}}}}$$

DFT calculations reveal that the rate-limiting step for CO_2_ reduction over Nb_2_O_5_ is the formation of *COOH intermediate. The adsorption of CO_2_ molecules displays spontaneity with a decrease in Gibbs free energy, reaffirming the inevitable chemisorption and activation of CO_2_ on Nb_2_O_5_. Based on the aforementioned analyses, the enhanced photocatalytic performance in CO_2_ reduction achieved by the In_2_O_3_/Nb_2_O_5_ S-scheme heterojunctions can be attributed to several pivotal factors: (i) the close interconnection between In_2_O_3_ and Nb_2_O_5_; (ii) the S-scheme-induced ultrafast photoelectron transfer at the heterojunction interfaces; (iii) the effective separation of powerful photoelectrons in the Nb_2_O_5_ CB and photoholes in the In_2_O_3_ VB; (iv) the prolonged lifetimes of charge carriers within the nanohybrids; and (v) the CO_2_ chemisorption and activation on Nb_2_O_5_.

In the liquid/solid photoreaction system, H_2_ production from H_2_O reduction competes with CO generation from CO_2_ reduction. To explore the influence of H_2_O content on product selectivity, different volumes of H_2_O were introduced into the reaction system. The results reveal an initial increase and subsequent decrease in CO production yield with the gradual addition of H_2_O (Fig. 6f). At a low H_2_O volume, fewer protons (H^+^) are available for CO_2_ reduction, resulting in poor performance. Conversely, excessive H_2_O in the reaction solvent diminishes the solubility of CO_2_ and impedes its activation on the catalyst. Notably, significant H_2_ production is absent in the absence of molecular catalysts and hole scavengers, regardless of H_2_O volume. Indeed, the high H_2_-evolution barrier over Nb_2_O_5_ poses a challenge for H_2_ production (Fig. 6e)^62^. Even if a trace amount of H_2_ is generated, it can be consumed by the residual O_2_ within the system, originating from input high-purity CO_2_ (99.999%), following an exothermic reaction (H_2_ + 1/2O_2_ → H_2_O, Δ*G* < 0). On the other hand, the CO selectivity is affected by the H_2_O content in the reaction system involving [Ru^II^(bpy)_3_]Cl_2_·6H_2_O and BIH, exhibiting a noticeable decrease when its volume exceeds 200 μL (Fig. 6g). This suggests that the input CO_2_/H_2_O can precisely tune the composition of the photoreduction products.

In a CO_2_ photoreduction system without hole sacrificial agents, two simultaneous processes govern the overall amount of O_2_ within the system: O_2_ generation from H_2_O photooxidation (H_2_O + 2 h^+^ → 1/2O_2_ + 2H^+^, OER) and its consumption through photoreduction (O_2_ + 4e^–^ + 4H^+^ → 2H_2_O, ORR). Over time, all the samples manifest a decline in O_2_ levels (Supplementary Fig. 28), indicating a higher rate of O_2_ consumption compared to its production. Free energy diagrams revel that the OER is not a spontaneous reaction for both In_2_O_3_ and Nb_2_O_5_, while the ORR is a spontaneous reaction (Supplementary Figs. 29 and 30), suggesting a preference for O_2_ consumption over its generation and, consequently, a substantial reduction in O_2_ quantity in the reaction system.

In summary, S-scheme In_2_O_3_/Nb_2_O_5_ hybrid nanofibers were synthesized using a facile one-step electrospinning method, establishing intimate phase contact for seamless charge carrier transport. Fs-TAS analyses confirmed the ultrafast electron transfer at the In_2_O_3_/Nb_2_O_5_ S-scheme heterojunction interface, involving the recombination of feeble photoelectrons in the In_2_O_3_ CB and holes in the Nb_2_O_5_ VB, while efficiently separating and preserving powerful photoelectrons in the Nb_2_O_5_ CB and holes in the In_2_O_3_ VB for participation in photoreactions. Both DFT calculations and CO_2_-TPD results demonstrated the efficient chemisorption and activation of CO_2_ molecules on the In_2_O_3_/Nb_2_O_5_ heterojunctions. Benefiting from the prolonged lifetimes driven by the rapid interfacial charge transfer and efficient CO_2_ activation on the catalyst, the optimized In_2_O_3_/Nb_2_O_5_ nanofibers exhibited enhanced CO_2_-reduction performance, yielding CO with an impressive output of up to 0.21 mmol g_active sites_^–1^ h^–1^ in the absence of any molecule cocatalyst or scavenger. This work highlights the potential of advanced fs-TAS techniques in exploring ultrafast charge transfer at S-scheme heterojunction interfaces.

## Methods

### Chemicals

All the chemicals are of analytical grade (AR) and were used without further purification. Indium nitrate hydrate (In(NO_3_)_3·_*x*H_2_O, 99.9%) and ammonium niobium oxalate (V) hydrate (C_4_H_4_NNbO_9·_*n*H_2_O, 99.9%) were purchased from Macklin Biochemical Technology Co., Ltd. (Shanghai, China). Polyvinylpyrrolidone (PVP, *M*_*W*_ = 1300,000) and tris(2,2′-bipyridyl)ruthenium(II) chloride hexahydrate ([Ru^II^(bpy)_3_]Cl_2_·6H_2_O, 98%) were purchased from Aladdin Biochemical Technology Co., Ltd. (Shanghai, China). N,N-dimethylformamide (DMF, AR) and acetonitrile (AR) were obtained from Sinopharm Chemical Reagent Co., Ltd. (Shanghai, China). 1,3-dimethyl-2-phenyl-2,3-dihydro-1H-benzo[d]imidazole (BIH) was synthesized according to our previous work^34^.

### Synthesis of In_2_O_3_/Nb_2_O_5_ (IN*x*) hybrid nanofibers

The In_2_O_3_/Nb_2_O_5_ hybrid nanofibers were synthesized via a one-step electrospinning method by mixing the precursors of both phases in the same electrospinning solution. Typically, 0.32 g of In(NO_3_)_3·_*x*H_2_O and 1.50 g of PVP were dissolved in 10 mL of DMF and stirred at room temperature until a clear solution was obtained. Simultaneously, 0.032 g of C_4_H_4_NNbO_9·_*n*H_2_O, corresponding to 10 wt.% of Nb_2_O_5_ relative to In_2_O_3_ (IN10), was dissolved in 1 mL of H_2_O. This solution was then added to the previously prepared one and stirred for 2 h. Subsequently, the viscous solution was loaded into a syringe equipped with a stainless-steel nozzle, positioned about 10 cm away from the collector. An electric potential of 20 kV was applied, and the solution was fed with a rate of 0.4 mL h^–1^. The collected nanofibrous mat was calcined at 600 °C for 2 h with a heating rate of 2 °C min^–1^ to completely remove the PVP, resulting in the formation of the In_2_O_3_/Nb_2_O_5_ nanofibers with a yield of over 90%. For comparison, In_2_O_3_/Nb_2_O_5_ heterojunctions with different Nb_2_O_5_ contents were synthesized by changing the amount of C_4_H_4_NNbO_9·_*n*H_2_O to 0.016 and 0.065 g, resulting in nominal weight percentages of 5 wt.% and 20 wt.% Nb_2_O_5_ relative to In_2_O_3_ (IN5 and IN20), respectively.

### Synthesis of pure In_2_O_3_ nanofibers

Pure In_2_O_3_ nanofibers were synthesized by dissolving 0.16 g of In(NO_3_)_3_·*x*H_2_O in 5 mL of DMF. Once the indium nitrate was completely dissolved, 0.75 g of PVP was added, and the mixture was stirred until the solution became clear. This solution was then loaded into a syringe equipped with a stainless-steel nozzle, positioned approximately 10 cm from the collector. A potential of 20 kV was applied, and the solution was fed at a rate of 0.4 mL h^–1^. The samples were collected and calcined at 600 °C for 2 h with a heating rate of 2 °C min^–1^ to obtain pure In_2_O_3_ nanofibers with a yield of approximately 90%.

### Synthesis of pure Nb_2_O_5_ nanorods

Typically, 0.08 g of C_4_H_4_NNbO_9·_*n*H_2_O was first dissolved in 1 mL of H_2_O. Once fully dissolved, 5 mL of DMF and 0.75 g of PVP were added while stirring until the solution was clear. This solution was then loaded into a syringe equipped with a stainless-steel nozzle, positioned about 10 cm from the collector. An electric potential of 20 kV was applied, and the solution was fed at a rate of 0.6 mL h^–1^. After calcining at 600 °C for 2 h with a heating rate of 2 °C min^–1^, pure Nb_2_O_5_ nanorods were obtained with a yield of over 90%.

### Photocatalytic CO_2_ reduction

The CO_2_ photoreduction was carried out in an online gas-closed system equipped with a gas-circulated pump (OLPCRS-2, Shanghai Boyi Scientific Instrument Co., Ltd.). Typically, 10 mg of photocatalysts, 30 mL of acetonitrile, and 100 μL of H_2_O were added into a glass reactor connected to the online system. The airtight system underwent complete evacuation using a vacuum pump. Then, ~60 kPa of high-purity CO_2_ (99.999%) gas was injected. After adsorption equilibrium, a 300 W Xe arc lamp (Microsolar 300 Xenon lamp source, Beijing Perfectlight, China) was used as the light source without any filter. The reaction system was maintained at 8 °C, controlled by cooling water. The gas chromatograph (GC-2030, Shimadzu Corp., Japan) equipped with barrier discharge ionization detector (BID) and a capillary column (Carboxen 1010 PLOT Capillary, 60 m × 0.53 mm) was employed to analyze the photocatalytic CO_2_ reduction products. For comparison, 2 mM of [Ru^II^(bpy)_3_]Cl_2_·6H_2_O and 10 mM of BIH were introduced into the photoreaction system, with other parameters unchanged. Regarding active sites, pure In_2_O_3_ employs itself as its active sites, while the IN*x* and pure Nb_2_O_5_ utilize the Nb_2_O_5_ as active sites. The value of *m*_active sites_ in all In_2_O_3_/Nb_2_O_5_ nanohybrids was determined based on the actual weight ratios of Nb_2_O_5_ within the composites (Supplementary Table 1). Given that the CB minimum and the VB maximum of the In_2_O_3_/Nb_2_O_5_ heterojunction are predominantly contributed by Nb 4*d* and O_In2O3_ 2*p* orbitals (Supplementary Fig. 31), it follows that CO_2_ photoreduction and H_2_O photooxidation occur specifically over the Nb and O_In2O3_ atoms, respectively^63^.

The isotope-labeling experiment was conducted using ^13^CO_2_ (isotope purity, 99%, and chemical purity, 99.9%) as the carbon source. The gas products were analyzed by gas chromatography-mass spectrometry (8890 GC System, 5977B GC/MSD, Agilent Technologies, USA) equipped with the column for detecting the reduction products (HP-MOLESIEVE). Helium was used as carrier gas. The temperatures of the injector and EI source were set to be 150 and 200 °C, respectively.

### Statistics and reproducibility

No statistical method was used to predetermine sample size. No data were excluded from the analyses. The experiments were not randomized, and we were not blinded to allocation during experiments and outcome assessment.

### Supplementary information

Supplementary Information

Peer Review File

### Source data

Source Data

## Data Availability

The source data underlying Figs. 1f, g, 2a–d, 3d–j, 4b–f, 5d–f, 6, and Supplementary Figs. 3–12, 13b-c, 14–17, 20, 21, 23–26, 28–31a are provided as a Source Data file, which is available in figshare with the identifier 10.6084/m9.figshare.25844110 and in the Source Data file. All data are available from the corresponding author on request. Source data are provided with this paper.
