# Supplementary material for: Ultrafast electron transfer at the In2O3/Nb2O5 S-scheme interface for CO2 photoreduction
Source: Nat Commun. 2024 Jun 5;15:4807. doi: 10.1038/s41467-024-49004-7 (PMC11153544; doi:10.1038/s41467-024-49004-7)
Supplement: Supplementary file 1 — Supplementary Information [file 41467_2024_49004_MOESM1_ESM.pdf]

Supplementary Information

Ultrafast Electron Transfer at the In_2O_3/Nb_2O_5 S-scheme Interface for CO_2 Photoreduction

Deng *et al.*

Supplementary Figures

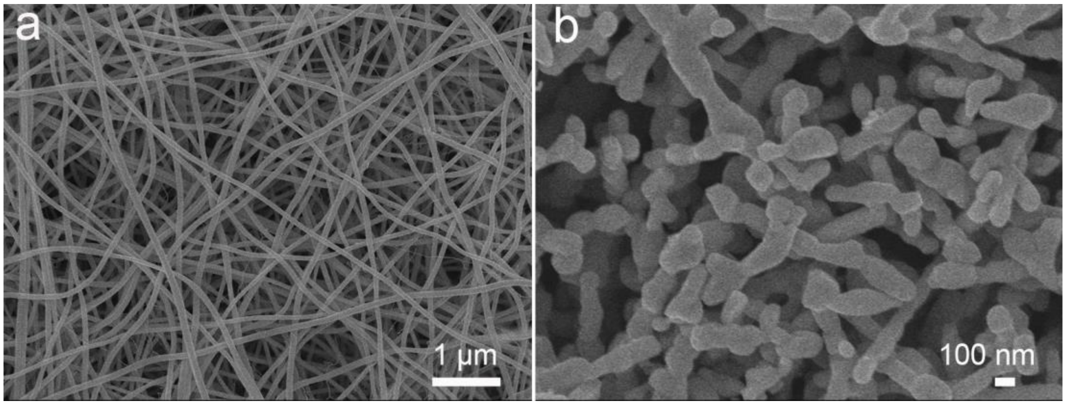

Supplementary Figure 1. FESEM images of **a** In_2O_3 nanofibers and **b** Nb_2O_5 nanorods.

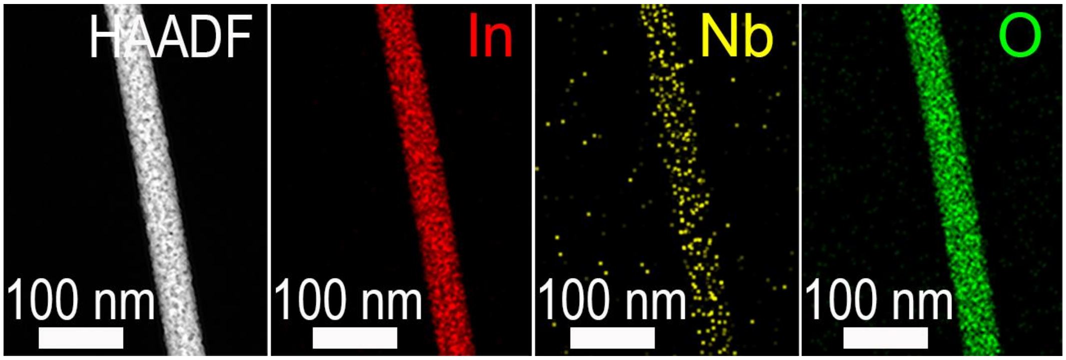

Supplementary Figure 2. High-angle annular dark-field (HAADF) image and EDX elemental mappings of In, Nb and O elements in IN10 at low magnification. IN10 represents the In_2O_3/Nb_2O_5 heterojunctions, where I and N denote In_2O_3 and Nb_2O_5, respectively, while *10* signifies the weight percentage of Nb_2O_5 relative to In_2O_3.

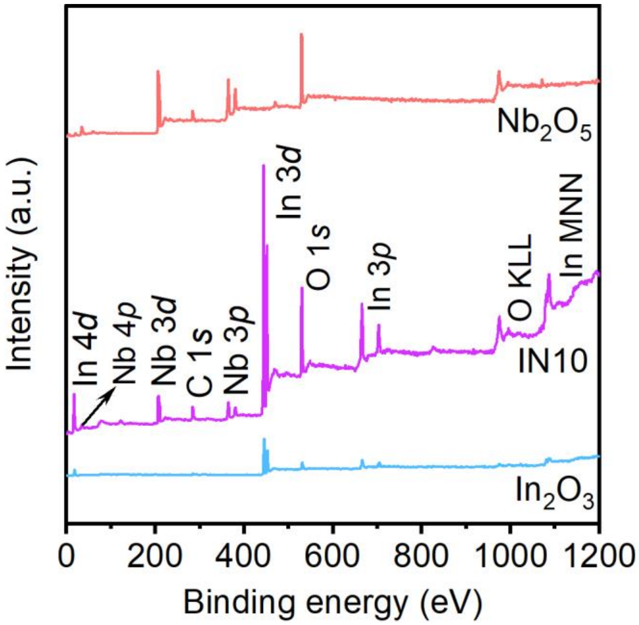

Supplementary Figure 3. The survey XPS spectra of In_2O_3, IN10, and Nb_2O_5. IN10 represents the In_2O_3/Nb_2O_5 heterojunctions, where I and N denote In_2O_3 and Nb_2O_5, respectively, while *10* signifies the weight percentage of Nb_2O_5 relative to In_2O_3.

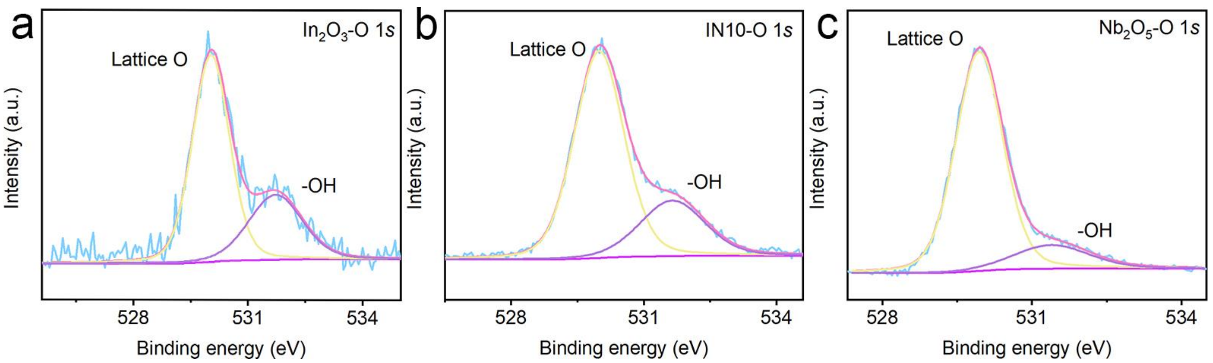

Supplementary Figure 4. The high-resolution XPS spectra of O 1*s*: a In_2O_3, **b** IN10, and **c** Nb_2O_5. IN10 represents the In_2O_3/Nb_2O_5 heterojunctions, where I and N denote In_2O_3 and Nb_2O_5, respectively, while *10* signifies the weight percentage of Nb_2O_5 relative to In_2O_3.

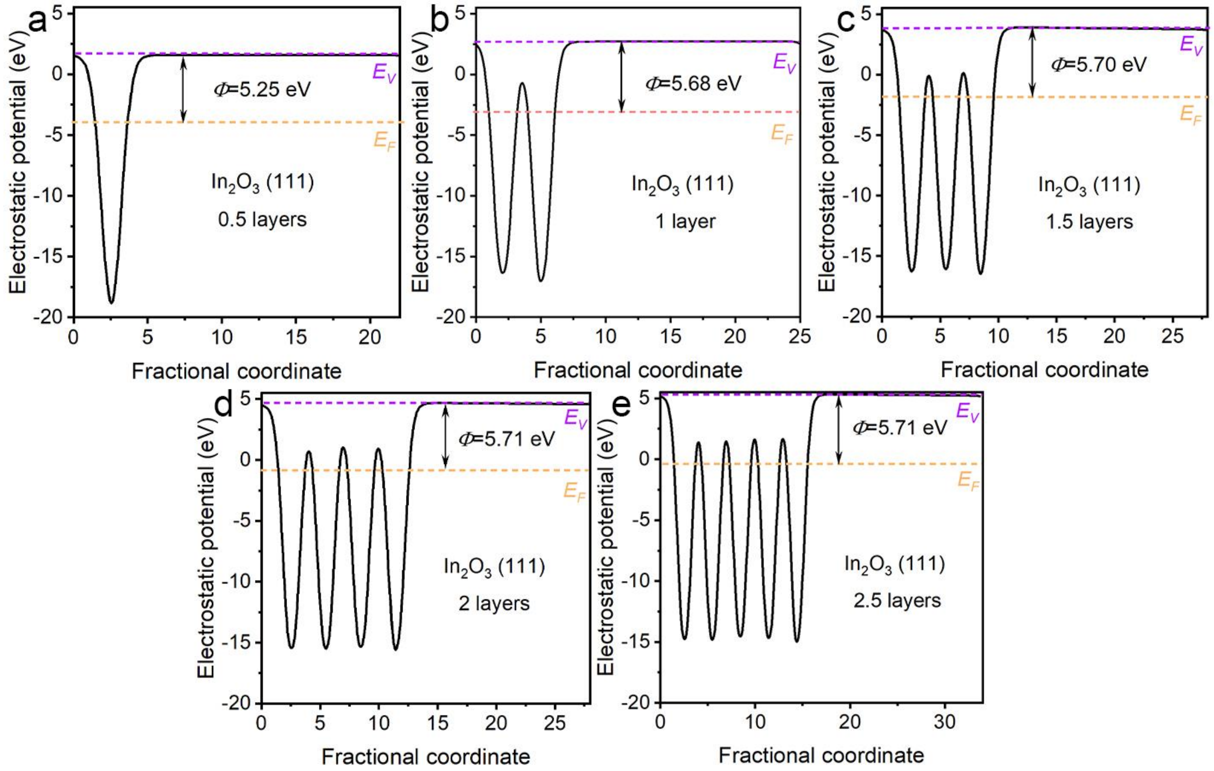

Supplementary Figure 5. Calculated electrostatic potentials of In_2O_3 (111) slabs with varying atomic layers: **a** 0.5 layers, **b** 1 layer, **c** 1.5 layers, **d** 2 layers, and **e** 2.5 layers.

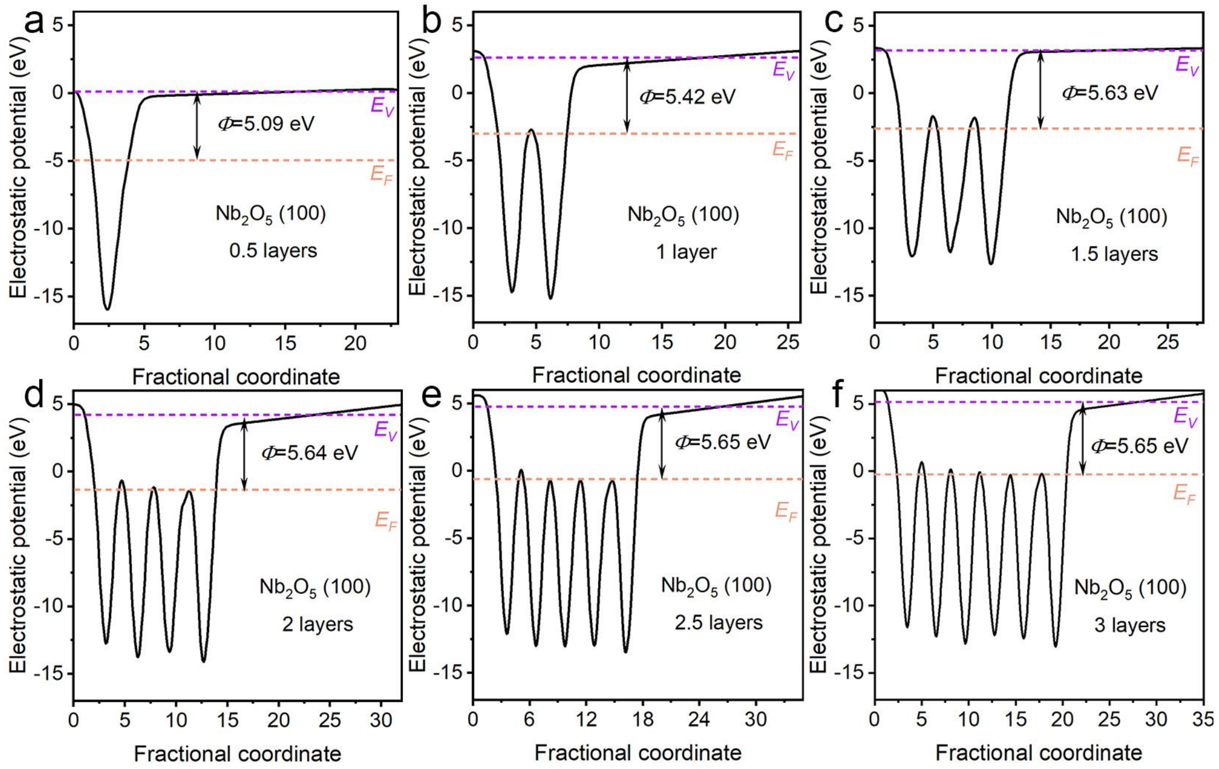

Supplementary Figure 6. Calculated electrostatic potentials of Nb_2O_5 (110) slabs with varying atomic layers: **a** 0.5 layers, **b** 1 layer, **c** 1.5 layers, **d** 2 layers, **e** 2.5 layers, and **f** 3 layers.

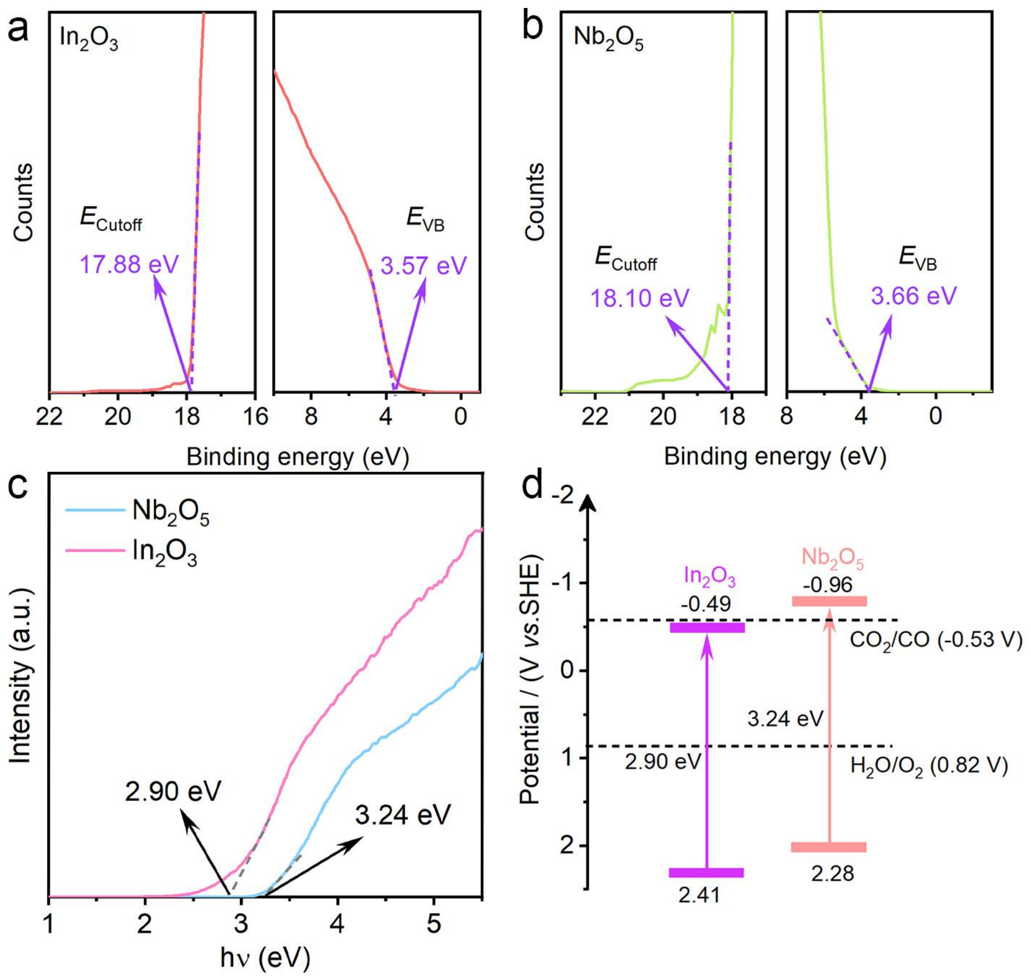

Supplementary Figure 7. Band structures of In_2O_3 and Nb_2O_5. UPS spectra of **a** In_2O_3 and **b** Nb_2O_5. **c** Kubelka-Munk energy curve plots and **d** band structures of In_2O_3 and Nb_2O_5.

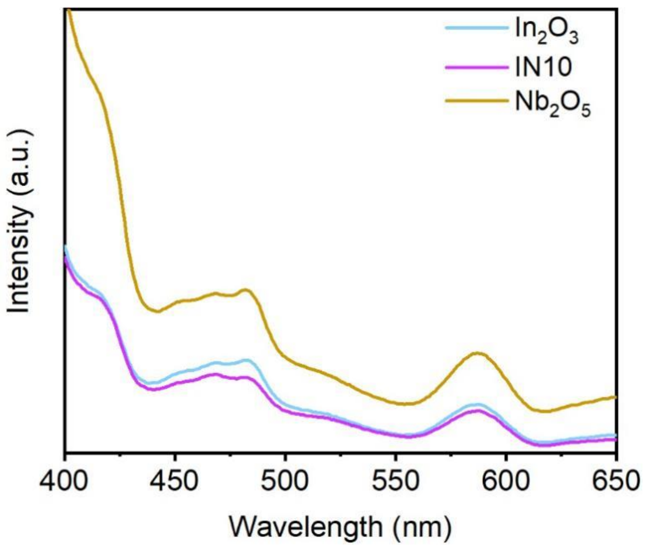

Supplementary Figure 8. PL spectra of In_2O_3, Nb_2O_5 and IN10. IN10 represents the In_2O_3/Nb_2O_5 heterojunctions, where I and N denote In_2O_3 and Nb_2O_5, respectively, while *10* signifies the weight percentage of Nb_2O_5 relative to In_2O_3.

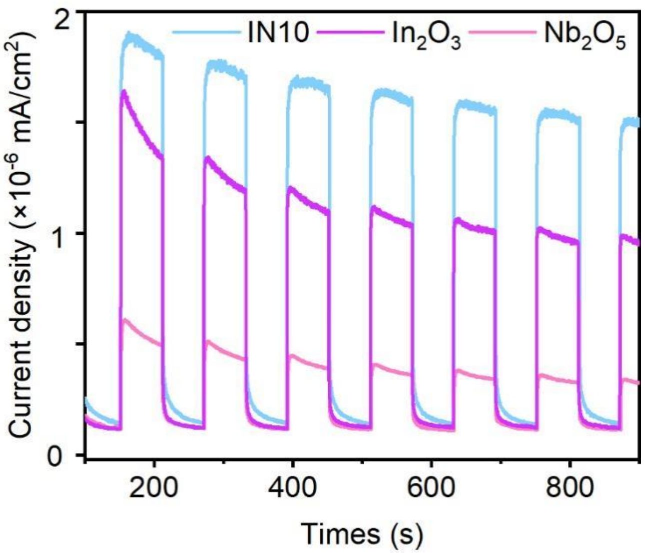

Supplementary Figure 9. Transient photocurrent response of In_2O_3, IN10 and Nb_2O_5. IN10 represents the In_2O_3/Nb_2O_5 heterojunctions, where I and N denote In_2O_3 and Nb_2O_5, respectively, while *10* signifies the weight percentage of Nb_2O_5 relative to In_2O_3.

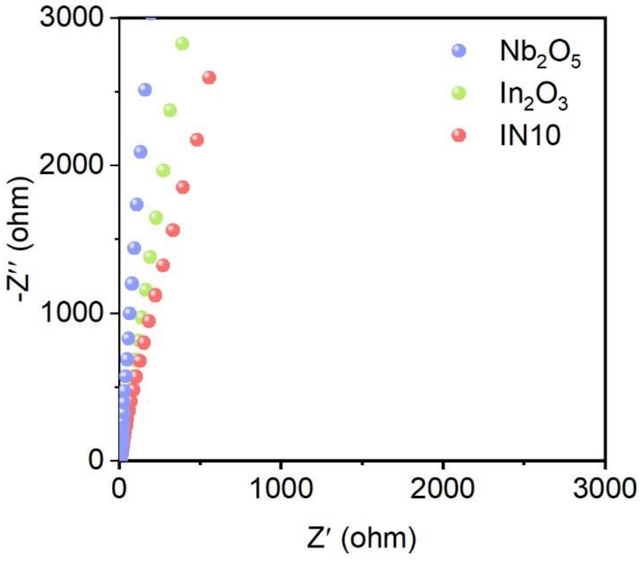

Supplementary Figure 10. Nyquist plots of In_2O_3, IN10 and Nb_2O_5. IN10 represents the In_2O_3/Nb_2O_5 heterojunctions, where I and N denote In_2O_3 and Nb_2O_5, respectively, while *10* signifies the weight percentage of Nb_2O_5 relative to In_2O_3.

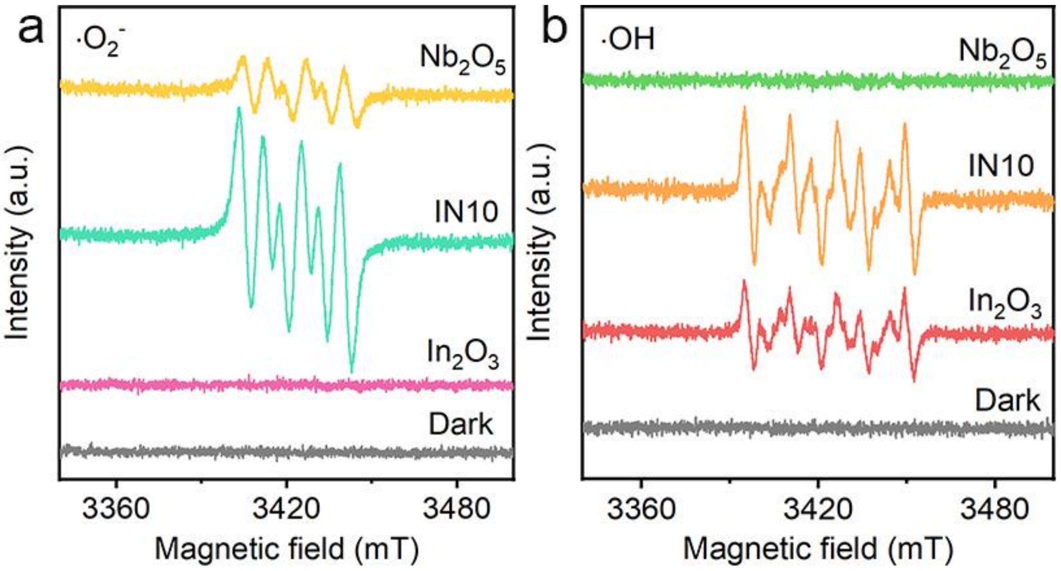

Supplementary Figure 11. EPR spectra of **a** DMPO-·O_2^- and **b** DMPO-·OH species in aqueous solution produced over In_2O_3, Nb_2O_5 and IN10. IN10 represents the In_2O_3/Nb_2O_5 heterojunctions, where I and N denote In_2O_3 and Nb_2O_5, respectively, while *10* signifies the weight percentage of Nb_2O_5 relative to In_2O_3.

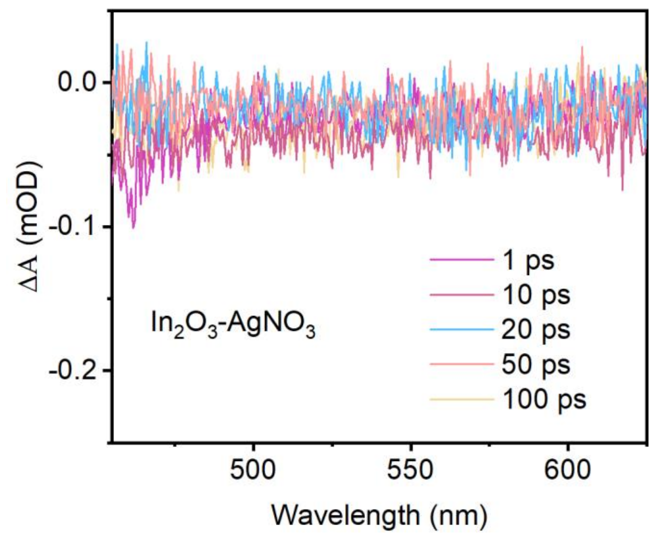

Supplementary Figure 12. The transient absorption spectra of pure In_2O_3 recorded following the addition of an electron scavenger ($AgNO_3$) and measured with 340 nm excitation.

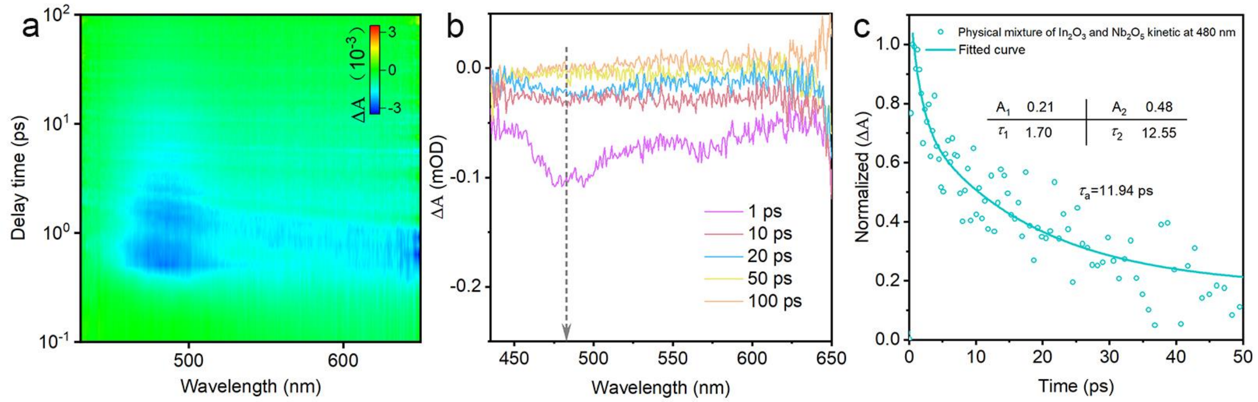

Supplementary Figure 13. The fs-TAS analysis of physically-mixed composite of In_2O_3 and Nb_2O_5. a The pseudocolor plot, and **b** transient absorption spectra recorded at indicated delay times measured with 340 nm excitation. **c** Corresponding kinetic decay curve at 480 nm within 50 ps in Ar.

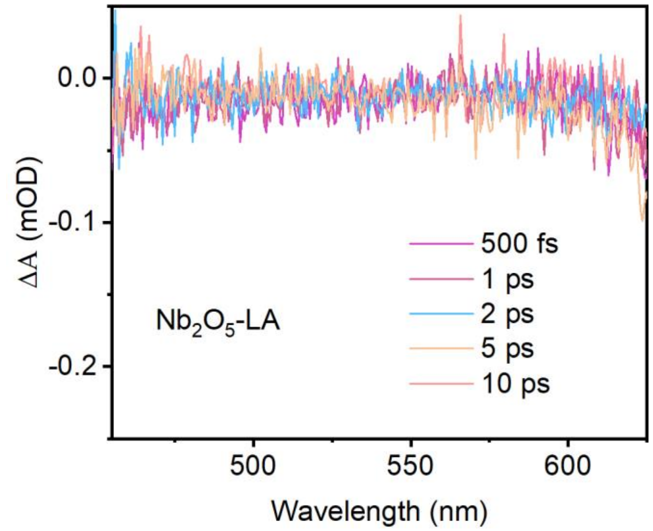

Supplementary Figure 14. The transient absorption spectra of pure Nb_2O_5 recorded following the addition of a hole scavenger (lactic acid, LA) and measured with 340 nm excitation.

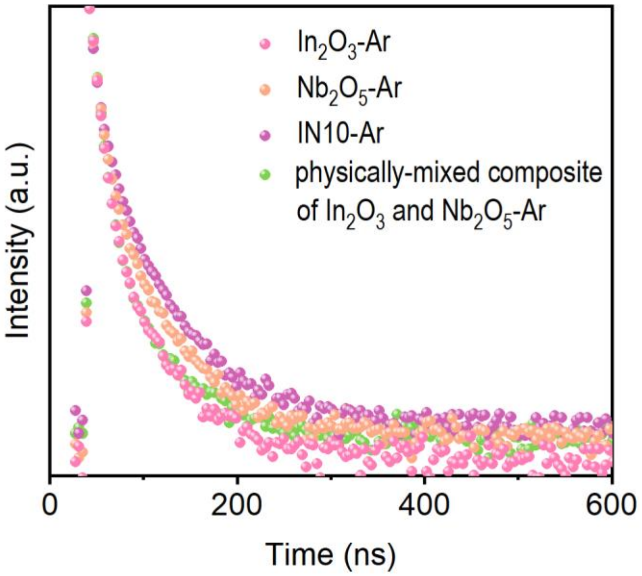

Supplementary Figure 15. TRPL spectra of In_2O_3, Nb_2O_5, IN10, as well as physically-mixed composite of In_2O_3 and Nb_2O_5 collected at an emission wavelength of 470 nm in an Ar atmosphere. IN10 represents the In_2O_3/Nb_2O_5 heterojunctions, where I and N denote In_2O_3 and Nb_2O_5, respectively, while *10* signifies the weight percentage of Nb_2O_5 relative to In_2O_3.

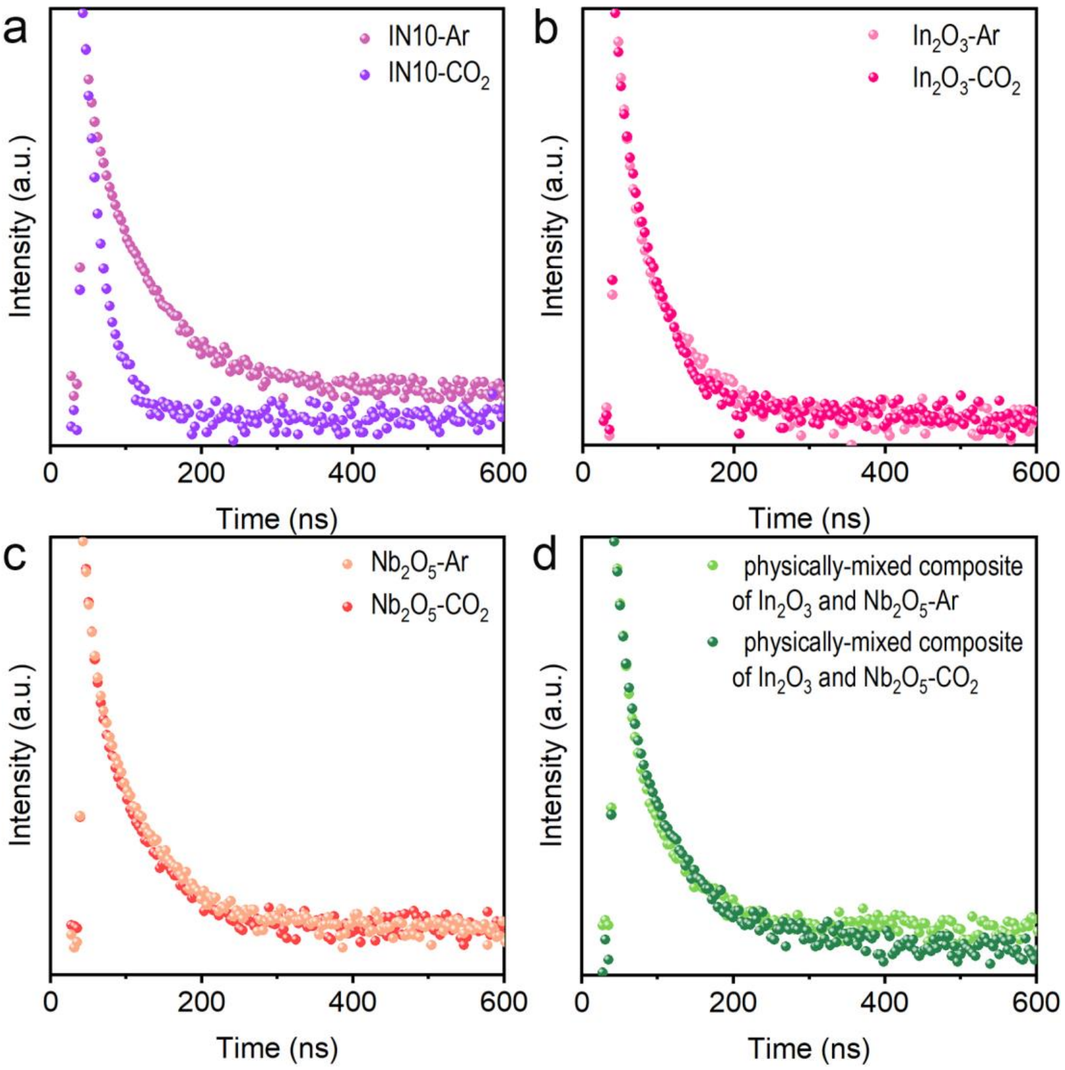

Supplementary Figure 16. In situ TRPL spectra collected at an emission wavelength of 470 nm in CO_2 atmospheres: **a** IN10, **b** In_2O_3, **c** Nb_2O_5, and **d** physically-mixed composite of In_2O_3 and Nb_2O_5. IN10 represents the In_2O_3/Nb_2O_5 heterojunctions, where I and N denote In_2O_3 and Nb_2O_5, respectively, while *10* signifies the weight percentage of Nb_2O_5 relative to In_2O_3.

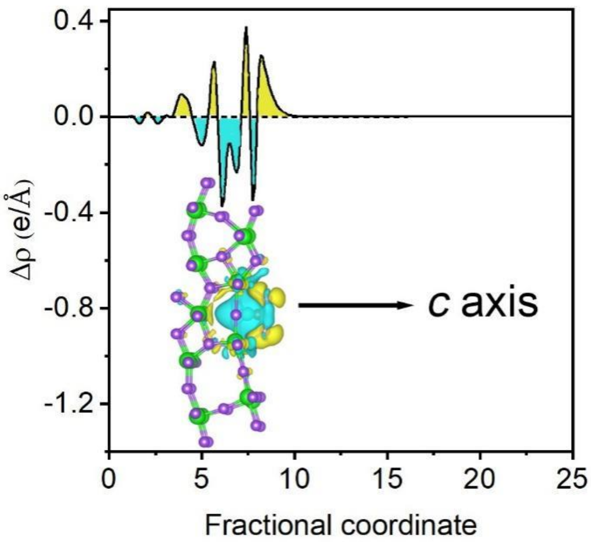

Supplementary Figure 17. Planar-averaged electron density difference $\Delta\rho$ of CO_2 absorbed on Nb_2O_5 surface. Cyan and yellow areas indicate electron depletion and accumulation, respectively.

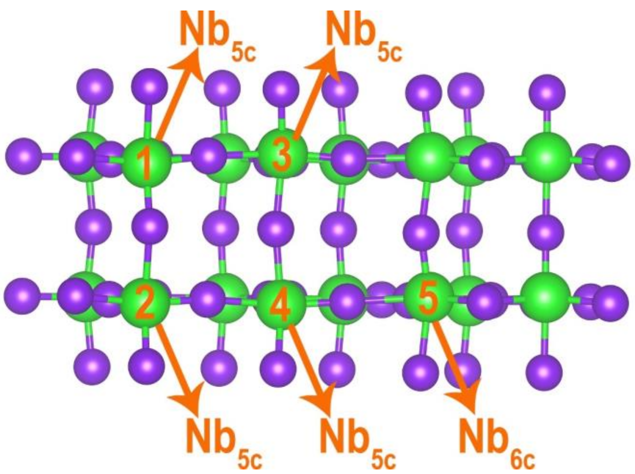

Supplementary Figure 18. Various kinds of equivalent adsorption sites of the Nb_2O_5 surface.

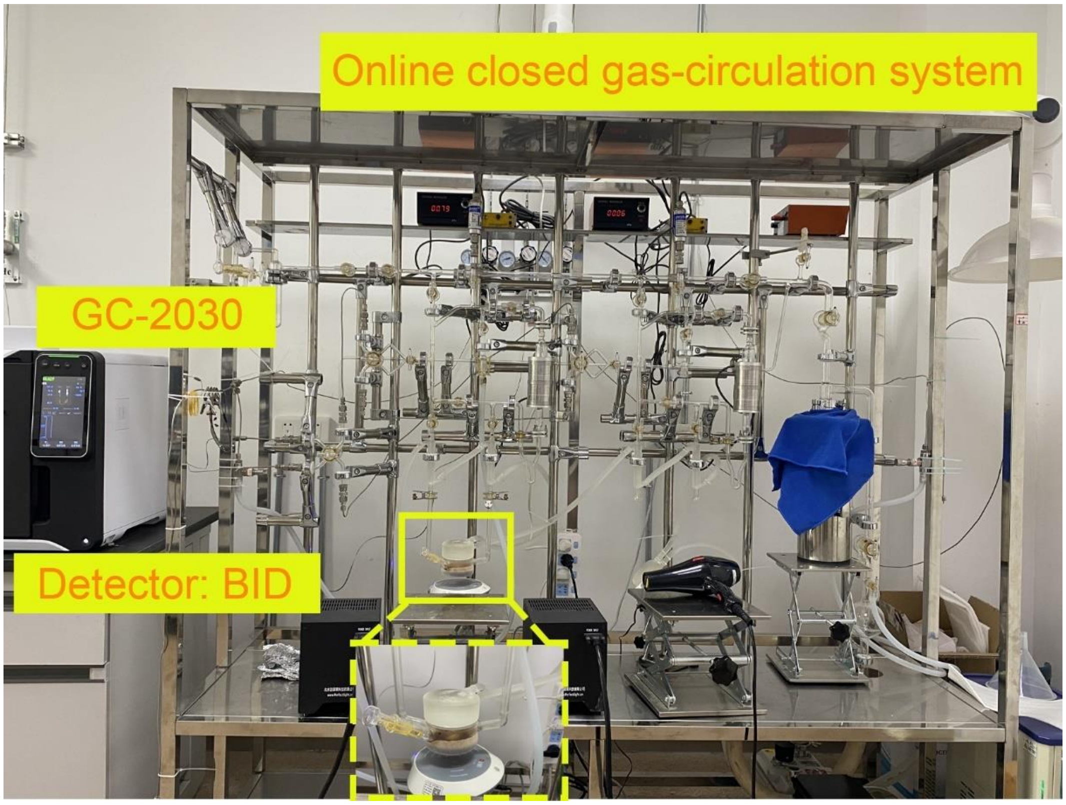

Supplementary Figure 19. The online closed gas-circulation system for CO_2 photoreduction.

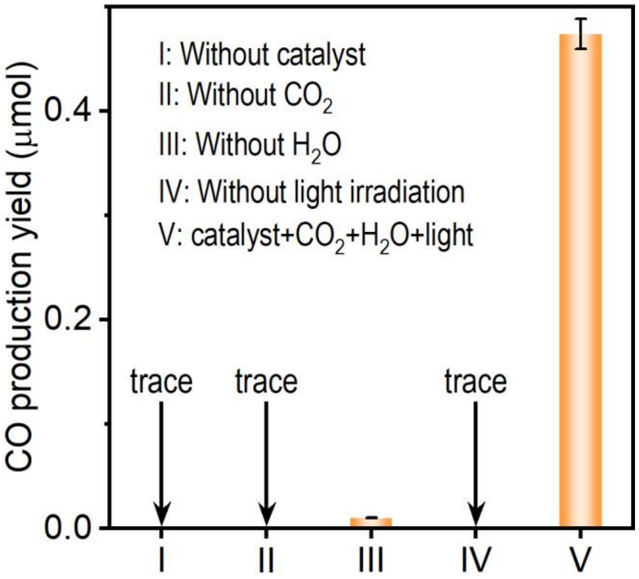

Supplementary Figure 20. Blank control experiments conducted under conditions without catalyst, CO_2, H_2O, and light irradiation, respectively. A trace amount of CO was detected without the introduction of H_2O, primarily attributed to residual moisture or vapor within in the solvent and ambient surroundings. The error bars (mean ± standard deviation) were obtained based on three independent photocatalytic experiments.

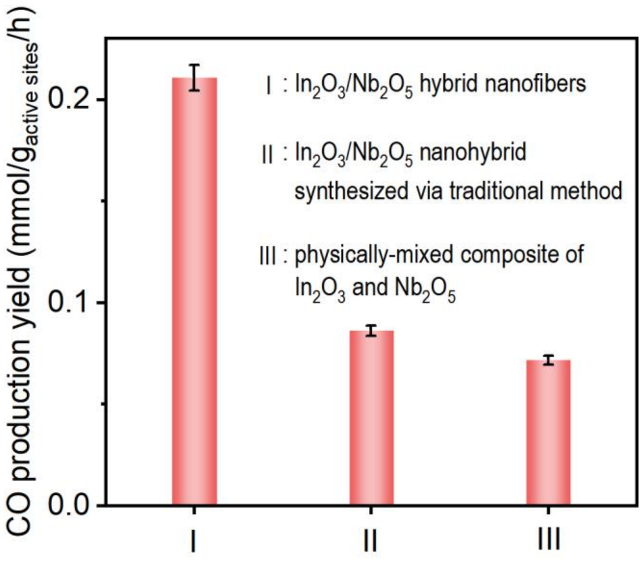

Supplementary Figure 21. The comparison of CO_2 photoreduction performance over various In_2O_3/Nb_2O_5 composites. The error bars (mean ± standard deviation) were obtained based on three independent photocatalytic experiments.

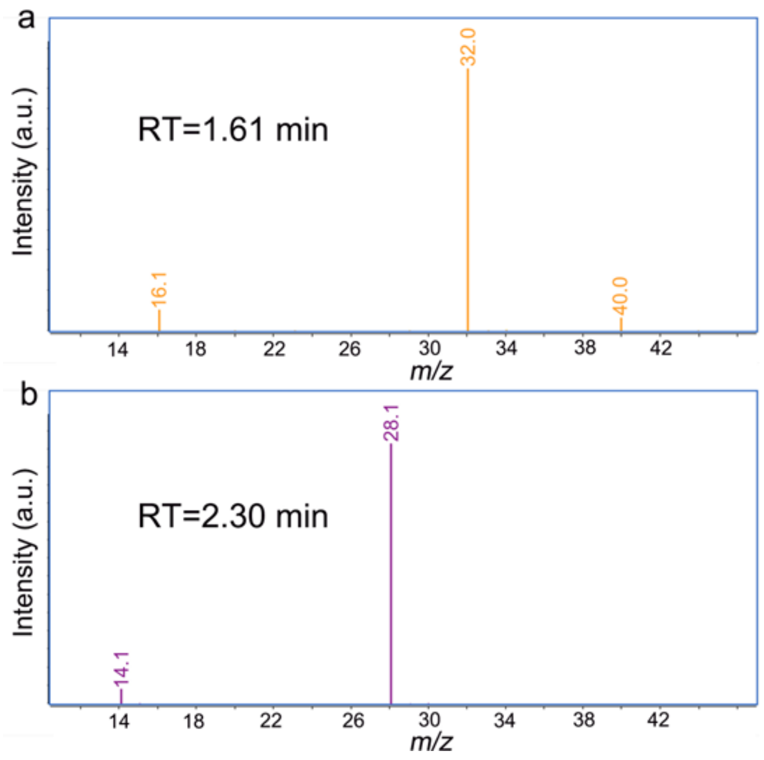

Supplementary Figure 22. Mass spectra of **a** O_2/Ar and **b** N_2 over IN10 in the photocatalytic reduction of $^{13}CO_2$. IN10 represents the In_2O_3/Nb_2O_5 heterojunctions, where I and N denote In_2O_3 and Nb_2O_5, respectively, while *10* signifies the weight percentage of Nb_2O_5 relative to In_2O_3.

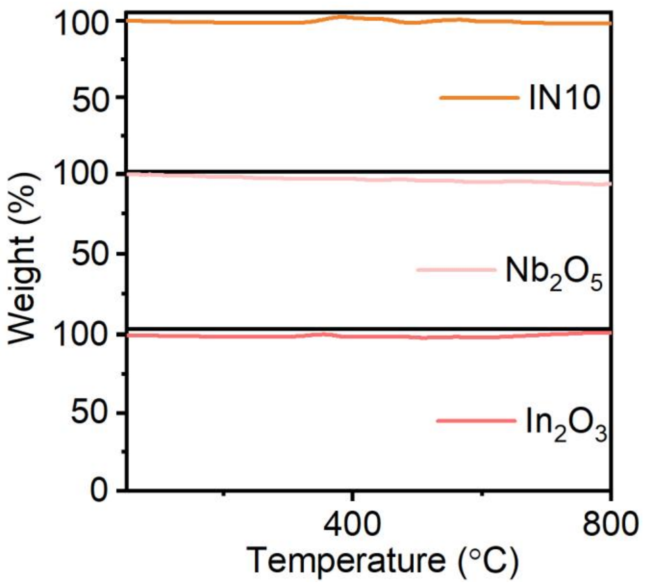

Supplementary Figure 23. Thermogravimetric (TGA) curves of pure In_2O_3, pristine Nb_2O_5 and In_2O_3/Nb_2O_5 nanohybrid (IN10). IN10 represents the In_2O_3/Nb_2O_5 heterojunctions, where I and N denote In_2O_3 and Nb_2O_5, respectively, while *10* signifies the weight percentage of Nb_2O_5 relative to In_2O_3.

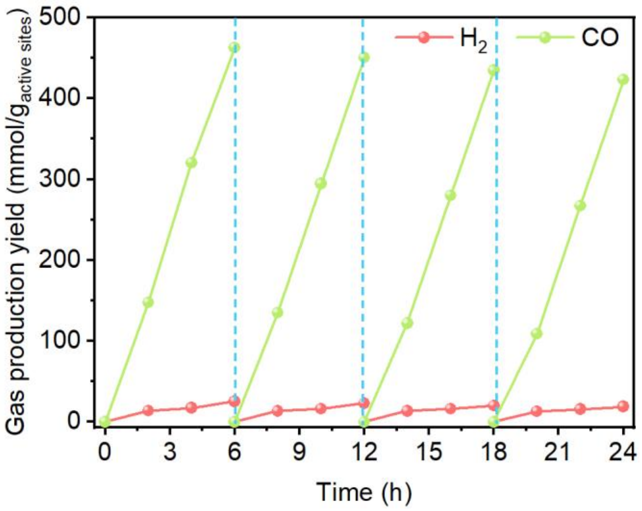

Supplementary Figure 24. Generation of products over IN10 in stability tests for four recycles with tris(2,2′-bipyridyl)ruthenium(II) chloride hexahydrate ($[Ru^{II}(bpy)_3]Cl_2{\cdot}6H_2O$) and 1,3-dimethyl-2-phenyl-2,3-dihydro-1H-benzo[d]imidazole (BIH) as the molecular catalyst and hole scavenger, respectively. IN10 represents the In_2O_3/Nb_2O_5 heterojunctions, where I and N denote In_2O_3 and Nb_2O_5, respectively, while *10* signifies the weight percentage of Nb_2O_5 relative to In_2O_3.

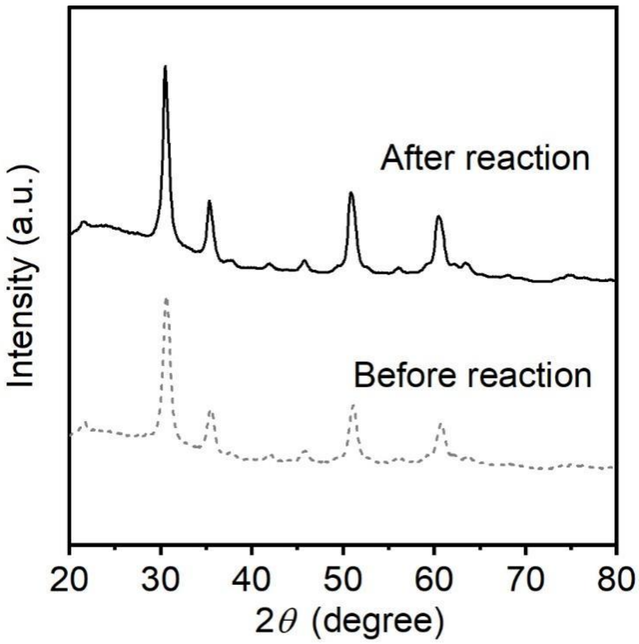

Supplementary Figure 25. The XRD patterns of IN10 before and after photoreactions. IN10 represents the In_2O_3/Nb_2O_5 heterojunctions, where I and N denote In_2O_3 and Nb_2O_5, respectively, while *10* signifies the weight percentage of Nb_2O_5 relative to In_2O_3.

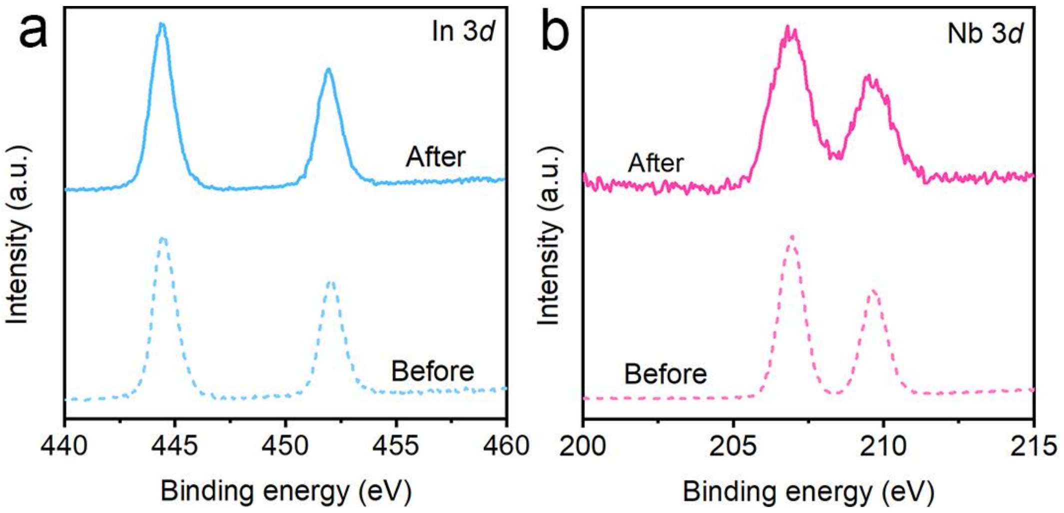

Supplementary Figure 26. The high-resolution XPS spectra of **a** In 3*d*, and **b** Nb 3*d* of IN10 before and after photoreaction. IN10 represents the In_2O_3/Nb_2O_5 heterojunctions, where I and N denote In_2O_3 and Nb_2O_5, respectively, while *10* signifies the weight percentage of Nb_2O_5 relative to In_2O_3.

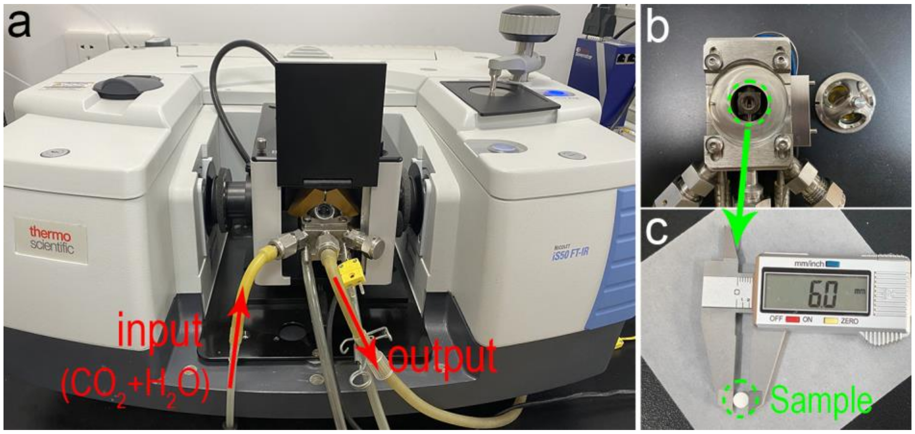

Supplementary Figure 27. Schematic illustration of the instrument and reaction cell for the in situ DRIFTS experiment. **a** Photo of the instrument and the gas flow diagram. **b** Photo of the reaction cell. **c** Photo of the sample.

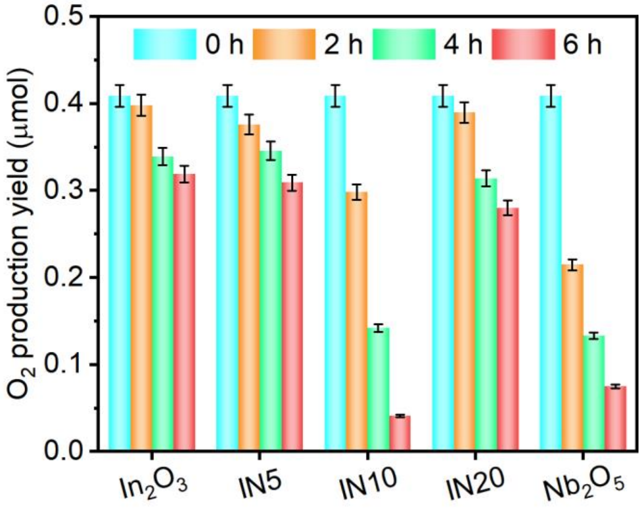

Supplementary Figure 28. Time course of the microscopic amount of O_2 within the system over In_2O_3, IN*x* and Nb_2O_5 under UV-visible light irradiation. IN*x* represents the In_2O_3/Nb_2O_5 heterojunctions, where I and N denote In_2O_3 and Nb_2O_5, respectively, while *x* signifies the weight percentage of Nb_2O_5 relative to In_2O_3. The error bars (mean ± standard deviation) were obtained based on three independent photocatalytic experiments.

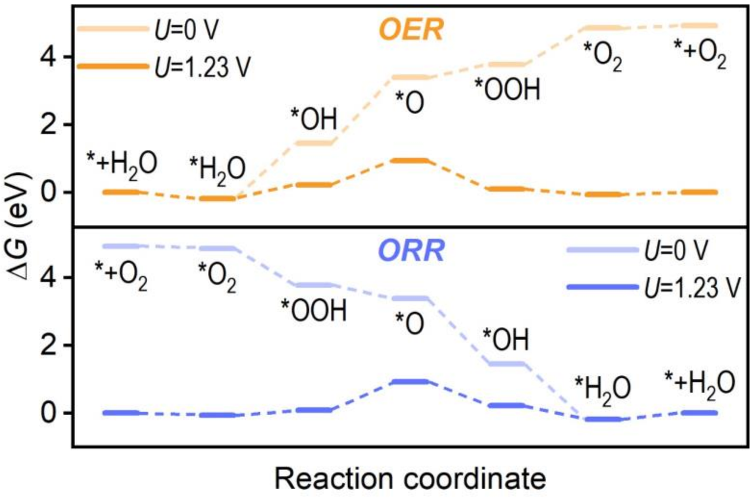

Supplementary Figure 29. Gibbs free energy diagrams for H_2O oxidation (OER) and O_2 reduction (ORR) over In_2O_3 at different electrode potential *U*.

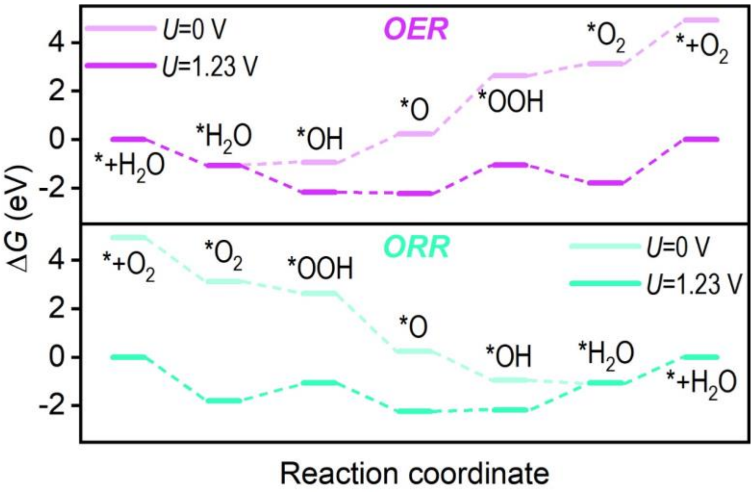

Supplementary Figure 30. Gibbs free energy diagrams for H_2O oxidation (OER) and O_2 reduction (ORR) over Nb_2O_5 at different electrode potential *U*.

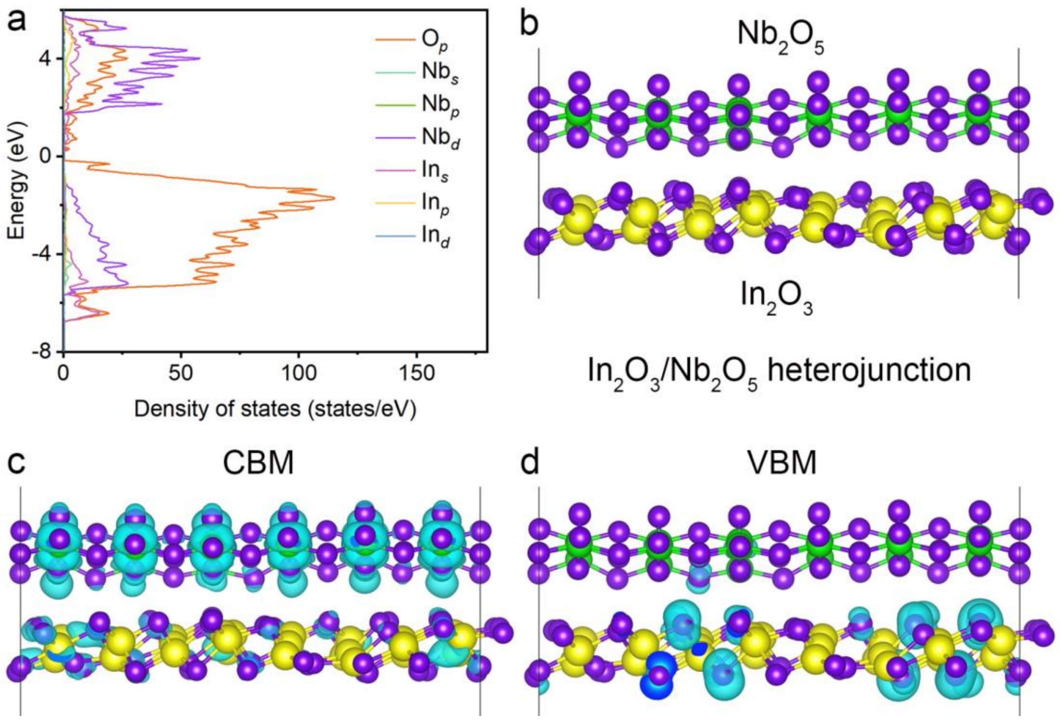

Supplementary Figure 31. Calculation of the In_2O_3/Nb_2O_5 heterojunction. a The projected density of states and **b** the optimized structure of the In_2O_3/Nb_2O_5 heterojunction. The band decomposed charged density of **c** the conduction band minimum (CBM) and **d** the valence band maximum (VBM) of the In_2O_3/Nb_2O_5 heterojunction. The Fermi level was set to zero. The yellow, green and violet spheres represent In, Nb and O atoms, respectively.

Supplementary Tables

Supplementary Table 1. The precise Nb_2O_5 content in all composites determined *via* ICP-AES.

Sample	$m_{(Nb2O5)}/m_{(In2O3)}$	wt.%(Nb_2O_5)
**IN5**	1.9%	1.86%
**IN10**	3.9%	3.75%
**IN20**	6.1%	5.75%

Supplementary Table 2. The calculated surface energy of various facets of In_2O_3.

Facet	Surface energy (J m^{-2})
**100**	2.02
**110**	1.06
**111**	0.74
**201**	1.65

Supplementary Table 3. The calculated surface energy of various facets of Nb_2O_5.

Facet	Surface energy (J m^{-2})
**001**	0.85
**010**	0.72
**100**	0.54
**110**	0.86
**101**	1.26
**011**	1.03
**111**	0.85

The surface energy γ is defined as the energy per unit area required to form the surface relative to the bulk. It is calculated using the following formula:

$$\gamma = \frac{U_{\text{slab}} - U_{\text{bulk}}}{2A} \qquad \text{(S1)}$$

Here, U_{slab} represents the total energy of the relaxed surface slab with a 20 Å vacuum region, U_{bulk} denotes the energy of the equivalent bulk In_2O_3 or Nb_2O_5 units, and A represents the surface area created on each side of the surface slab. During the calculation of surface energy, the middle layer of the surface slab was fixed, while the top and bottom layers were allowed to relax.

Supplementary Table 4. The fitted lifetimes obtained from kinetic decay curves at 480 nm for In_2O_3, IN5 and IN10 under different atmospheres. IN*x* represents the In_2O_3/Nb_2O_5 heterojunctions, where I and N denote In_2O_3 and Nb_2O_5, respectively, while *x* signifies the weight percentage of Nb_2O_5 relative to In_2O_3.

**Sample**	A_1	τ_1 **(ps)**	A_2	τ_2 **(ps)**	A_3	τ_3 **(ps)**
**In_2O_3-Ar**	0.21	1.60	0.52	12.69	-	-
**IN5-Ar**	0.37	1.03	0.07	4.90	0.33	12.87
**IN10-Ar**	0.23	0.33	0.25	1.84	0.37	10.30
**IN10-CO_2**	0.21	4.48E-4	0.36	1.02	0.35	8.79

Supplementary Table 5. The atomic Bader charge of each atom of the adsorbed CO_2 on the Nb_2O_5 surface.

Atoms	Bader valence electron charges	Charge transfer (relative to atom)
Nb1	10.3664	2.6336
Nb2	10.3435	2.6565
Nb3	10.3861	2.6139
Nb4	10.3214	2.6786
Nb5	10.342	2.658
Nb6	10.2981	2.7019
Nb7	10.3446	2.6554
Nb8	10.3675	2.6325
Nb9	10.4731	2.5269
Nb10	10.3136	2.6864
Nb11	10.3106	2.6894
Nb12	10.3345	2.6655
Nb13	10.3082	2.6918
Nb14	10.335	2.665
Nb15	10.3386	2.6614
Nb16	10.3394	2.6606
O1	7.0757	-1.0757
O2	7.0138	-1.0138
O3	7.0822	-1.0822
O4	7.0422	-1.0422
O5	7.0534	-1.0534
O6	7.0529	-1.0529
O7	7.0916	-1.0916
O8	7.0435	-1.0435
O9	7.1248	-1.1248
O10	7.0232	-1.0232
O11	7.0643	-1.0643
O12	7.063	-1.063
O13	7.0558	-1.0558
O14	7.0523	-1.0523
O15	7.1102	-1.1102
O16	7.0829	-1.0829
O17	6.9908	-0.9908
O18	7.0322	-1.0322
O19	7.1125	-1.1125
O20	7.0693	-1.0693

O21	6.9596	-0.9596
O22	7.0173	-1.0173
O23	7.1203	-1.1203
O24	7.1128	-1.1128
O25	7.1526	-1.1526
O26	7.1693	-1.1693
O27	7.0652	-1.0652
O28	7.0669	-1.0669
O29	7.0713	-1.0713
O30	7.0729	-1.0729
O31	7.1509	-1.1509
O32	7.1724	-1.1724
O33	7.0973	-1.0973
O34	7.0664	-1.0664
O35	6.9617	-0.9617
O36	7.0342	-1.0342
O37	7.0469	-1.0469
O38	7.1086	-1.1086
O39	6.8577	-0.8577
O40	6.7876	-0.7876
Nb17	10.3641	2.6359
Nb18	10.3374	2.6626
Nb19	10.3804	2.6196
Nb20	10.3428	2.6572
Nb21	10.344	2.656
Nb22	10.3077	2.6923
Nb23	10.3723	2.6277
Nb24	10.3844	2.6156
Nb25	10.4665	2.5335
Nb26	10.3438	2.6562
Nb27	10.3258	2.6742
Nb28	10.3394	2.6606
Nb29	10.3085	2.6915
Nb30	10.3533	2.6467
Nb31	10.3415	2.6585
Nb32	10.3407	2.6593
O41	7.0757	-1.0757
O42	7.0113	-1.0113
O43	7.0803	-1.0803
O44	7.0728	-1.0728
O45	7.0537	-1.0537

O46	7.0412	-1.0412
O47	7.091	-1.091
O48	7.0616	-1.0616
O49	7.1251	-1.1251
O50	7.0026	-1.0026
O51	7.0625	-1.0625
O52	7.0669	-1.0669
O53	7.0556	-1.0556
O54	7.0528	-1.0528
O55	7.1092	-1.1092
O56	7.0835	-1.0835
O57	6.9911	-0.9911
O58	7.0322	-1.0322
O59	7.0946	-1.0946
O60	7.0804	-1.0804
O61	6.9581	-0.9581
O62	7.0299	-1.0299
O63	7.1086	-1.1086
O64	7.1196	-1.1196
O65	7.1406	-1.1406
O66	7.1057	-1.1057
O67	7.0673	-1.0673
O68	7.0642	-1.0642
O69	7.0741	-1.0741
O70	7.0661	-1.0661
O71	7.1503	-1.1503
O72	7.1803	-1.1803
O73	7.0984	-1.0984
O74	7.0589	-1.0589
O75	6.9588	-0.9588
O76	7.0391	-1.0391
O77	7.0557	-1.0557
O78	7.0813	-1.0813
O79	6.8496	-0.8496
O80	6.789	-0.789
C	1.8639	2.1361
O81	7.1186	-1.1186
O82	7.1195	-1.1195

Supplementary Table 6. The adsorption energy (*E_{ads}*) of CO_2 molecules at various sites on the Nb_2O_5.

**Adsorption sites**	***E_{ads}* (eV)**
Nb_{5c} (#1) and Nb_{5c} (#2) *via* two O-Nb linkages	-1.90
Nb_{5c} (#3) and Nb_{5c} (#4) *via* two O-Nb linkages	-1.75
Nb_{5c} (#2) and Nb_{5c} (#4) *via* two O-Nb linkages	-2.05
Nb_{5c} (#4) and Nb_{6c} (#5) *via* two O-Nb linkages	-2.08
O atom	-0.78

Note: the labeling of Nb atoms is depicted in Supplementary Fig. 18.

Supplementary Methods

Characterization. X-Ray diffraction (XRD) patterns of the obtained photocatalysts were recorded using a Shimadzu XRD-6100 X-ray diffractometer (Japan) with Cu *K*α radiation. Field emission scanning electron microscope (FESEM) images were obtained from JSM 7500F (JEOL, Japan). Transmission electron microscopy (TEM), high-resolution TEM (HRTEM), and the corresponding energy-dispersive X-ray spectroscopy (EDX) mappings were conducted with a Thermal Fisher Talos F200X instrument. The light absorption spectra of the prepared samples were measured using a Shimadzu UV-2600 UV-visible spectrophotometer (Japan). The chemical compositions and elemental valence states of the samples were analyzed by X-ray photoelectron spectroscopy (XPS) using a Thermo ESCALAB 250Xi XPS spectrometer system (USA) with 300 W Al *K*α radiation. In situ irradiation XPS measurements were conducted under the same conditions with the addition of light irradiation. Ultraviolet photoelectron spectroscopy (UPS) measurements were performed on a Thermo Fisher ESCALAB 250Xi instrument with the He I radiation ($h\nu$ = 21.22 eV) source. The band structure (E_{CBM} and E_{VBM}) of In_2O_3 and Nb_2O_5 was calculated using the following empirical formulas:

$$E_{\mathrm{VBM}} = h\nu - (E_{\mathrm{cutoff}} - E_{\mathrm{VB}}) \qquad \text{(S2)}$$

$$E_{\mathrm{CBM}} = E_{\mathrm{g}} - E_{\mathrm{VBM}} \qquad \text{(S3)}$$

where $h\nu$ and E_g represent the photon energy and bandgap respectively. E_{cutoff} and E_{VB} denote the cutoff edge and the valence-band spectra, respectively, obtained from the UPS spectra. The detailed calculation process is listed as follows:

$$E_{VBM}(In_2O_3, \text{vs. } E_V) = 21.22 - (17.88 - 3.57) = 6.91 \text{ V}$$
$$E_{VBM}(In_2O_3, \text{vs. SHE}) = 6.91 - 4.5 = 2.41 \text{ V} \quad \text{(S4)}$$
$$E_{CBM}(In_2O_3, \text{vs. SHE}) = 2.41 - 2.90 = -0.49 \text{ V}$$

$$E_{VBM}(Nb_2O_5, \text{vs. } E_V) = 21.22 - (18.10 - 3.66) = 6.78 \text{ V}$$
$$E_{VBM}(Nb_2O_5, \text{vs. SHE}) = 6.78 - 4.5 = 2.28 \text{ V} \quad \text{(S5)}$$
$$E_{CBM}(Nb_2O_5, \text{vs. SHE}) = 2.28 - 3.24 = -0.96 \text{ V}$$

In situ diffuse reflectance infrared Fourier transform spectra (DRIFTS) were acquired on the Nicolet iS50 spectrometer (Thermo Scientific, USA) equipped with a specialized reactor (Supplementary Fig. 27). Prior to measurement, samples were compressed into cylinder shapes with a diameter of 0.6 cm under 10 MPa. The experimental procedure involved two sequential stages in a continuous-flow mode. Initially, CO_2 was purged into the chamber with saturated water vapor at a flow rate of 20 mL min^{-1} for 60 minutes in the absence of light to explore the CO_2 adsorption on the photocatalyst. Subsequently, a 365-nm LED light was activated for another 60 minutes to investigate the photoreaction intermediates. Electrochemical measurements were carried out using a standard three-electrode system (CHI660C, China) with an electrolyte containing 0.5 M Na_2SO_4 in a total volume of 50 mL. The counter electrode was a Pt wire (1.0 cm^2), and the reference electrode was Ag/AgCl (filled with saturated KCl). The working electrode was prepared by dispersing 20 mg of catalysts in 1.0 mL of ethanol and 10 μL of Nafion solution under grinding for 20 minutes. The mixture was then coated onto the conductive surface of FTO glasses with an active area of about 1.0 cm^2 and dried in an oven at 60 °C overnight.

Photoluminescence (PL) spectra of the samples were analyzed by a fluorescence spectrophotometer (F-4700, Hitachi, Japan) with the excitation wavelength at 325 nm. Time-

resolved photoluminescence (TRPL) spectra was collected on a fluorescence lifetime spectrophotometer (FLS 1000, Edinburgh, UK) using a laser as the light source with an excitation wavelength of 375 nm and the maximum average power of 5 mW. The average lifetime (τ_a) can be calculated according to the following equation:

$$\tau_a = \frac{A_1\tau_1^2 + A_2\tau_2^2 + A_3\tau_3^2}{A_1\tau_1 + A_2\tau_2 + A_3\tau_3} \qquad \text{(S6)}$$

where τ_1, τ_2 and τ_3 stand for the lifetime of radiative, non-radiative and energy-transfer process, respectively. A_1, A_2 and A_3 are the preexponential factors of decay curves. Femtosecond transient absorption spectroscopy (fs-TAS) analysis of a pump-probe system (Helios, ultrafast system) with a maximum delay of about 8 ns was performed under ambient conditions using a motorized optical delay line. A pump pulse of 340 nm (sample average power of about 80 μW) was output from an ultrafast optical parametric amplifier (OPera Solo) excited using a regenerative amplifier (Coherent Astrella, 800 nm, 35 fs, 5 mJ, 1 kHz) and injected with a mode-locked Ti:Sapphire oscillator (Coherent Vitara, 800 nm, 80 MHz) for injection and an LBO laser (Coherent evolutic-50c, 1 kHz system) for pumping. A small number of 800 nm femtosecond pulses generated by the regenerative amplifier were utilized to pump the sapphire crystal, producing a 350-800 nm white light continuum as a probe pulse. Samples were dispersed in acetonitrile with 5 mg of catalyst. The mixture was added into quartz cuvettes with a path length of 2 mm. Cuvettes were sealed using rubber septa caps and degassed with Ar or CO_2 for 10 minutes.

The decay curves obtained from the TA spectra were fitted by the following multi-exponential equation:

$$I_{(t)} = I_{(0)} + \sum_{i=1}^{n} A_i \exp(-t/\tau_i) \qquad \text{(S7)}$$

Where I_0 represents the baseline correction value, and t is the probe time delay. A_i and τ_i are amplitudes and decay times, respectively.

Computational details. The periodic DFT calculations were performed by using Vienna Ab-initio Simulation Package (VASP). The exchange correlation between electrons was treated using the generalized gradient approximation (GGA) in the Pardew-Burke-Ernzerhof (PBE) form. A (1×1) supercell of In_2O_3 (111) and a (2×2) supercell of Nb_2O_5 (100) surface slabs were adopted, where the half bottom atomic layers were fixed for all calculations. To avoid interactions between periodic structures, ~20 Å vacuum space was inserted. The cutoff energy was chosen at 520 eV, and the Brillouin zone was sampled using *k*-point spacing of 0.02 $Å^{-1}$. The convergence thresholds for energy and atomic forces were set as 10^{-5} eV and 0.02 eV $Å^{-1}$, respectively. The van der Waals interaction between adsorbate and slab was corrected with the DFT-D3(BJ) method. The free energy (G) for each isolated or adsorbed molecule was calculated at 298.15 K with zero-point energy (ZPE) correction. The formula is $G = E + \text{ZPE} - TS$, where E is the electronic energy calculated with VASP, ZPE is the zero-point energy and TS is the entropy contribution. The work function (Φ) of In_2O_3 (111) and Nb_2O_5 (100) slabs were calculated according to the formula of $\Phi = E_V - E_F$, where E_V and E_F stand for the vacuum energy level and Fermi level of the slabs, respectively.
